# Beyond Chloride Brines: Variable Metabolomic Responses in the Anaerobic Organism *Yersinia intermedia* MASE-LG-1 to NaCl and MgSO_4_ at Identical Water Activity

**DOI:** 10.3389/fmicb.2018.00335

**Published:** 2018-02-27

**Authors:** Petra Schwendner, Maria Bohmeier, Petra Rettberg, Kristina Beblo-Vranesevic, Frédéric Gaboyer, Christine Moissl-Eichinger, Alexandra K. Perras, Pauline Vannier, Viggó T. Marteinsson, Laura Garcia-Descalzo, Felipe Gómez, Moustafa Malki, Ricardo Amils, Frances Westall, Andreas Riedo, Euan P. Monaghan, Pascale Ehrenfreund, Patricia Cabezas, Nicolas Walter, Charles Cockell

**Affiliations:** ^1^School of Physics and Astronomy, UK Center for Astrobiology, University of Edinburgh, Edinburgh, United Kingdom; ^2^Radiation Biology Department, German Aerospace Center (DLR), Institute of Aerospace Medicine, Cologne, Germany; ^3^Centre de Biophysique Moléculaire, Centre National de la Recherche Scientifique, Orléans, France; ^4^Department of Internal Medicine, Medical University of Graz, Graz, Austria; ^5^BioTechMed-Graz, Graz, Austria; ^6^Department of Microbiology and Archaea, University of Regensburg, Regensburg, Germany; ^7^MATIS - Prokaria, Reykjavík, Iceland; ^8^Faculty of Food Science and Nutrition, University of Iceland, Reykjavik, Iceland; ^9^Instituto Nacional de Técnica Aeroespacial - Centro de Astrobiología, Madrid, Spain; ^10^Centro de Biología Molecular Severo Ochoa (CBMSO, CSIC-UAM), Universidad Autónoma de Madrid, Madrid, Spain; ^11^Leiden Observatory, Universiteit Leiden, Leiden, Netherlands; ^12^Space Policy Institute, George Washington University, Washington, DC, United States; ^13^European Science Foundation, Strasbourg, France

**Keywords:** sodium chloride, magnesium sulfate, metabolome, compatible solutes, stress response

## Abstract

Growth in sodium chloride (NaCl) is known to induce stress in non-halophilic microorganisms leading to effects on the microbial metabolism and cell structure. Microorganisms have evolved a number of adaptations, both structural and metabolic, to counteract osmotic stress. These strategies are well-understood for organisms in NaCl-rich brines such as the accumulation of certain organic solutes (known as either compatible solutes or osmolytes). Less well studied are responses to ionic environments such as sulfate-rich brines which are prevalent on Earth but can also be found on Mars. In this paper, we investigated the global metabolic response of the anaerobic bacterium *Yersinia intermedia* MASE-LG-1 to osmotic salt stress induced by either magnesium sulfate (MgSO_4_) or NaCl at the same water activity (0.975). Using a non-targeted mass spectrometry approach, the intensity of hundreds of metabolites was measured. The compatible solutes L-asparagine and sucrose were found to be increased in both MgSO_4_ and NaCl compared to the control sample, suggesting a similar osmotic response to different ionic environments. We were able to demonstrate that *Yersinia intermedia* MASE-LG-1 accumulated a range of other compatible solutes. However, we also found the global metabolic responses, especially with regard to amino acid metabolism and carbohydrate metabolism, to be salt-specific, thus, suggesting ion-specific regulation of specific metabolic pathways.

## Introduction

The concentrations of dissolved salt in the environment can vary substantially. Understanding how microorganisms respond to different salt environments has applications to investigating microorganisms in hypersaline environments on Earth (Kaye and Baross, 2004) with implications for biomedical and biotechnical research and the potential survivability of microorganisms in salty extraterrestrial environments, such as brines on Mars (Carr, 1996; Osterloo et al., 2008; Ojha et al., 2015). In the latter case, research is primarily driven by an interest in determining habitability of extraterrestrial environments and in the fate of terrestrial contaminants transferred to that planet on spacecraft (planetary protection).

Most research on the effects of salt on microbial communities has focused on NaCl-rich environments. Aside from the occurrence of NaCl-rich brines, sulfate-rich bodies of water are found on Earth (Eugster and Hardie, 1978; Renaut and Long, 1989; Nesbitt, 1990; Yakimov et al., 2015). Sulfates are also known to be an important component of the Martian soil (Bibring et al., 2007; Hynek et al., 2015). In addition to our poor knowledge of how microorganisms acclimate to different ionic environments, we also know very little about how anaerobic microorganisms adapt to such extremes. Yet in the subsurface, evaporitic deposits are often anoxic.

Studies of microbial adaptations to high salt have focused on aerobic strains or were performed under oxic conditions (reviewed in Csonka, 1989; Kempf and Bremer, 1998; Roberts, 2005). Although some studies of osmotolerance on anaerobic microorganisms have been done (Beblo-Vranesevic et al., 2017a), we still do not know how microorganisms survive in brines of different salts nor their individual metabolic response to either of the salts. Research work using anaerobic microorganisms to make a comparison between metabolic responses to different salts would considerably improve our understanding of microbial growth in a wide range of natural environments.

Fluctuations in salt concentrations cause changes in water availability by creating either hyper- or hypo-osmotic conditions leading to osmotic stress for cells. Under hyperosmotic conditions, i.e., increase in salt concentration and without active regulation, the cell may shrink due to rapid efflux of water until the internal and external water activity is balanced. This can ultimately lead to immediate cell death due to plasmolysis (Poolman and Glaasker, 1998). However, some microorganisms have evolved evolutionarily well-conserved strategies that enable them to adapt to and cope with changes in the external osmolarity (Csonka, 1989). Although the individual mechanisms of these strategies vary and are species-specific, two common strategies are distinguished.

One strategy involves alterations in the membrane composition which includes both modifications in the phospholipid composition and fatty acid saturation to better cope with changes of the turgor pressure. The other is to re-establish the osmotic balance. The latter is grouped into two basic mechanisms: (i) Accumulation of inorganic ions such as K^+^, Mg^2+^, or Ca^2+^. This adaptation is mainly found in halophilic archaea and halotolerant bacteria (Oren, 2008). (ii) Exclusion of salts from the cytoplasm and synthesis and/or accumulation of organic osmolytes, so-called compatible solutes or osmoprotectants (Brown, 1976). These molecules include amino acids and their derivatives, sugars and sugar alcohols (Csonka, 1989). Their accumulation leads to the protection of proteins and membranes against the damages caused by high concentrations of inorganic ions.

To avoid any adverse effect, the osmolytes must be compatible with the physiology of the cell, i.e., they neither interfere with enzymatic activity, nor with DNA replication, nor with DNA–protein interactions (Kempf and Bremer, 1998). These solutes, which can be either synthesized *de novo* by the cell or accumulated from the medium, are usually neutral molecules that can be accumulated to molar levels. For most species the intake of compatible solutes from the medium is energetically more favourable than their synthesis, which is energetically very costly (Martin et al., 1999), more so for large organic molecules such as disaccharides, than for small molecules such as glycine betaine or ectoine (Oren, 1999). Therefore, microorganisms only use a limited number of compounds to cope with salt stress which are species-specific.

One way to unravel the biochemical responses of organisms to stress is to use metabolomics. The metabolome is the set of all metabolites present in cells at a given time, comprised of hundreds of chemically diverse and small molecules. The metabolites are often involved in several pathways. An untargeted metabolomics approach aims to study the entire set of metabolites within an organism by providing a snap-shot of the entire microbial metabolism at a specific point of time and under specific conditions. This technique is a powerful tool to unravel discrete changes in metabolic pathways and abundances of metabolites, allowing therefore a better understanding of biological responses to changes in environmental conditions (Nicholson and Lindon, 2008).

In this study we investigated the effect of salt stress on the facultative anaerobic *Yersinia intermedia* strain MASE-LG-1 (DSMZ 102845), a microorganism isolated in the framework of the EU MASE (Mars Analogues for Space Exploration) project (Cockell et al., 2017). This strain originates from lake Grænavatn (63 53.07′ N, 22 3.70′ W), a maar type explosion crater, with a pH value of around 2 and a mean temperature of 4°C. Its physiology and resistance to Mars relevant stresses have been previously studied (Beblo-Vranesevic et al., 2017b). We used metabolomics to investigate the effects at the same water activity of two different salts, NaCl and MgSO_4_. For the first time, this study provides insight not only into the global understanding of the response to osmotic stress caused by NaCl, but also focuses on the effects MgSO_4_. To understand how different salt environments would affect an anaerobic microorganism, we analysed metabolomics data to characterize stress molecular responses. We were able to identify metabolites associated with adaptation to these two different salts. These data have allowed us to identify the similarities and differences in microbial responses to NaCl- and MgSO_4_-containing fluids.

## Materials and methods

### Strain and cultivation conditions

*Yersinia intermedia* MASE-LG-1 strain was isolated in the framework of the MASE (Mars Analogues for Space Exploration) project (Cockell et al., 2017). For cultivation, the cells were grown under strictly anoxic conditions in a specifically designed MASE I medium (Cockell et al., 2017). Recipe per litre: NH_4_Cl 0.5 g, NaHCO_3_ 0.2 g, NaH_2_PO_4_ 0.06 g, 1 × Wolfe's Minerals 10 ml (Balch et al., 1979), 1 × Wolfe's Vitamins 10 ml (Balch et al., 1979), cysteine-HCl 0.5 g. The pH was adjusted to pH 7. The strain specific supplements are: 0.01% (w/v) KNO_3_, 0.01% (w/v) C-Org-Mix (50 ml stock solution: yeast extract 0.5 g, peptone 0.5 g, brain heart infusion 0.5 g, meat extract 0.5 g, 1 × Wolfe's Vitamins 500 μl). The medium was portioned (20 ml each) into 120 ml serum bottles and gassed with 1 bar N_2_/CO_2_ (80/20 vol/vol) (Miller and Wolin, 1974). The incubation was carried out at 30°C with shaking at 50 rpm.

### Inducing salt stress

*Yersinia intermedia* MASE-LG-1 was tested for its growth limits in saline conditions using sodium chloride (NaCl concentration was varied from 0 to 1.1 M in 0.1 increments, and at 1.5 M), magnesium sulfate (MgSO_4_ × 7 H_2_O concentration was varied from 0 to 1.1 M in 0.1 increments, and at 1.5 and 2 M). The isolates were transferred to the MASE medium supplemented with the specific salts and subjected in triplicates to the conditions. Growth was observed by microscopy and cell numbers were determined using a Thoma cell counting chamber.

### Water activity measurements

A HydroPalm HP23-AW water activity meter (Rotronic AG, Switzerland) was used to determine the water activity (a_w_) of the growth medium supplemented with salts and for making adjustments to reach the same water activity in the samples used for metabolomics. Prior to measurement, the meter was calibrated to five points (a_w_ = 0.325, 0.595, 0.755, 0.845, and 0.935) using saturated calibration standards (MgCl_2_, NH_4_NO_3_, NaCl, KCl, and KH_2_PO_4_, respectively) prepared as described by Winston and Bates (1960). Five millilitres of each medium were measured three times, and the results were found to be within ±0.003 a_w_.

### Water activity of growth

Although different brines may have different ionic strengths and compositions, water activity is thought to be one of the primary physico-chemical factors that sets a hard limit to life (Williams and Hallsworth, 2009; Stevenson et al., 2015). Therefore, in the experiments reported here, we poised the NaCl and MgSO_4_ fluids that we used at the same water activity to allow for a comparison of the metabolic responses of different brines without being influenced by the water activity value.

### Metabolite extraction

Three biological replicates were used in parallel. Once the stresses were applied and the maximum time of incubation was reached, the samples were quenched immediately under anoxic conditions. To capture the true state of metabolism under stressed conditions the enzyme activities have to be arrested instantaneously as some of the metabolites can have a very high turnover rate. Therefore, the samples were transferred from the inoculation vial with an ice cold syringe to a Falcon tube that was immediately shock-frozen with liquid nitrogen. To maintain an anoxic atmosphere and to avoid any changes due to the exposure to oxygen, this was done under an argon atmosphere. This step was followed by centrifugation at 4°C for 10 min at 1,000 g. The supernatant was removed. The pellet was resuspended in 1 ml supernatant, transferred to an Eppendorf tube and centrifuged again at 4°C for 5 min at 2,500 g. The supernatant was removed completely and the cell pellet was sonicated for 10 min at 4°C after adding 200 μl of chloroform/methanol/water (1:3:1 ratio; vol/vol/vol). The samples were vortexed under cooled (4°C) conditions for 1 h and centrifuged at 4°C for 3 min at 13,000 g. One hundred and eighty microliters of supernatant was transferred into a new vial and stored at −80°C until analysis. An additional sample of the extraction solvent (>200 μl) was obtained to allow removal of contaminants at the data-analysis stage. A pooled sample containing 10 μl from each sample analysed was used as a quality control sample in the LC-MS procedure.

### Liquid chromatography–mass spectrometry (LC-MS)

Hydrophilic interaction liquid chromatography (HILIC) was carried out on a Dionex UltiMate 3000 RSLC system (Thermo Fisher Scientific, Hemel Hempstead, UK) using a ZIC-pHILIC column (150 × 4.6 mm, 5 μm column, Merck Sequant). The column was maintained at 30°C and samples were eluted with a linear gradient (20 mM ammonium carbonate in water, A and acetonitrile, B) over 26 min at a flow rate of 0.3 ml/min as follows (time in minutes, % A, % B): 0, 20, 80; 15, 80, 20; 15, 95, 5; 17, 95, 5; 17, 20, 80; and 24, 20, 80. The injection volume was 10 μl and samples were maintained at 4°C prior to injection. For the MS analysis, a Thermo Orbitrap Exactive (Thermo Fisher Scientific) was operated in polarity switching mode and the MS settings were as follows: Resolution 50,000; AGC 106; m/z range 70–1,400; sheath gas 40; Auxiliary gas 5; sweep gas 1; Probe temperature 150°C and Capillary temperature 275°C. For positive mode ionisation: source voltage +4.5 kV, capillary voltage +50 V, tube voltage +70 kV, skimmer voltage +20 V. For negative mode ionisation: source voltage-3.5 kV, capillary voltage-50 V, tube voltage −70 V, skimmer voltage −20 V. Mass calibration was performed for each polarity immediately prior to each analysis batch. The calibration mass range was extended to cover small metabolites by inclusion of low-mass contaminants with the standard Thermo calmix masses (below m/z 1400), C_2_H_6_NO_2_ for positive ion electrospray ionisation (PIESI) mode (m/z 76.0393) and C_3_H_5_O_3_ for negative ion electrospray ionisation (NIESI) mode (m/z 89.0244). To enhance calibration stability, lock-mass correction was also applied to each analytical run using these ubiquitous low-mass contaminants.

### Data processing

We obtained metabolic profiles from three replicate cultures of *Y. intermedia* MASE-LG-1 grown under three different conditions. These are control (shown as “C”), salt stressed in MgSO_4_ (shown as “MgSO_4_”) or in NaCl (shown as “NaCl”). Instrument files (.raw) were processed as described in Creek et al. (2012). Briefly, the data set was converted to positive and negative ionisation mode mzXML files. These files were then analysed using the XCMS/mzMatch/IDEOM pipeline to produce the IDEOM file (Tautenhahn et al., 2008; Scheltema et al., 2011; Creek et al., 2012). IDEOM is a Microsoft Excel template that enables automated data processing to remove noise (estimated for 80% of peaks) from LC-MS metabolomics datasets. Metabolite identification was achieved by matching the accurate mass and retention time of the noise-corrected peaks to metabolites in the database. This database not only includes all likely metabolites from biological databases, but also the retention times for authentic standards carried during the run and a retention time prediction model. Once all the corrections were performed, the IDEOM file lists identified and rejected peaks with their corresponding confidence scores. Univariate statistics for mean values, relative intensity, Standard deviation, and Student's *t*-test were calculated automatically in Excel. Multivariate statistics were run in the R environment. Heatmaps were created using an algorithm that scaled each metabolite and then created the spectrum range based on the most intense compound throughout the list. Besides the IDEOM pipeline, the PiMP (Polyomics Metabolomics Pipeline) environment, an integrated, web-enabled tool for LC-MS data analysis and visualization was used. The peaks for each sample were called and retention time corrected using the OBI-Warp (Prince and Marcotte, 2006) algorithm. PiMP allows mapping the annotated metabolites to organism-specific metabolic networks from various databases (KEGG Kanehisa and Goto, 2000, BioCyc) and visualization thereof. The selection of significant different peaks was made based on adjusted *p*-values (Benjamini and Hochberg corrected) with a maximum of 0.05. Currently, the mapping and calculation of the library coverage table includes information from every organism available in the MetExplore database as no filter fallowing a search for a specific organism can be applied.

## Results

In this study, we aimed at deciphering metabolic modulations to osmotic stress on the intracellular metabolism of *Yersinia intermedia* MASE-LG-1 that were induced by the two different salts compared to control conditions.

### *Yersinia intermedia* MASE-LG-1 can grow in a broad range of NaCl and MgSO_4_ concentrations

**Figure 1:**
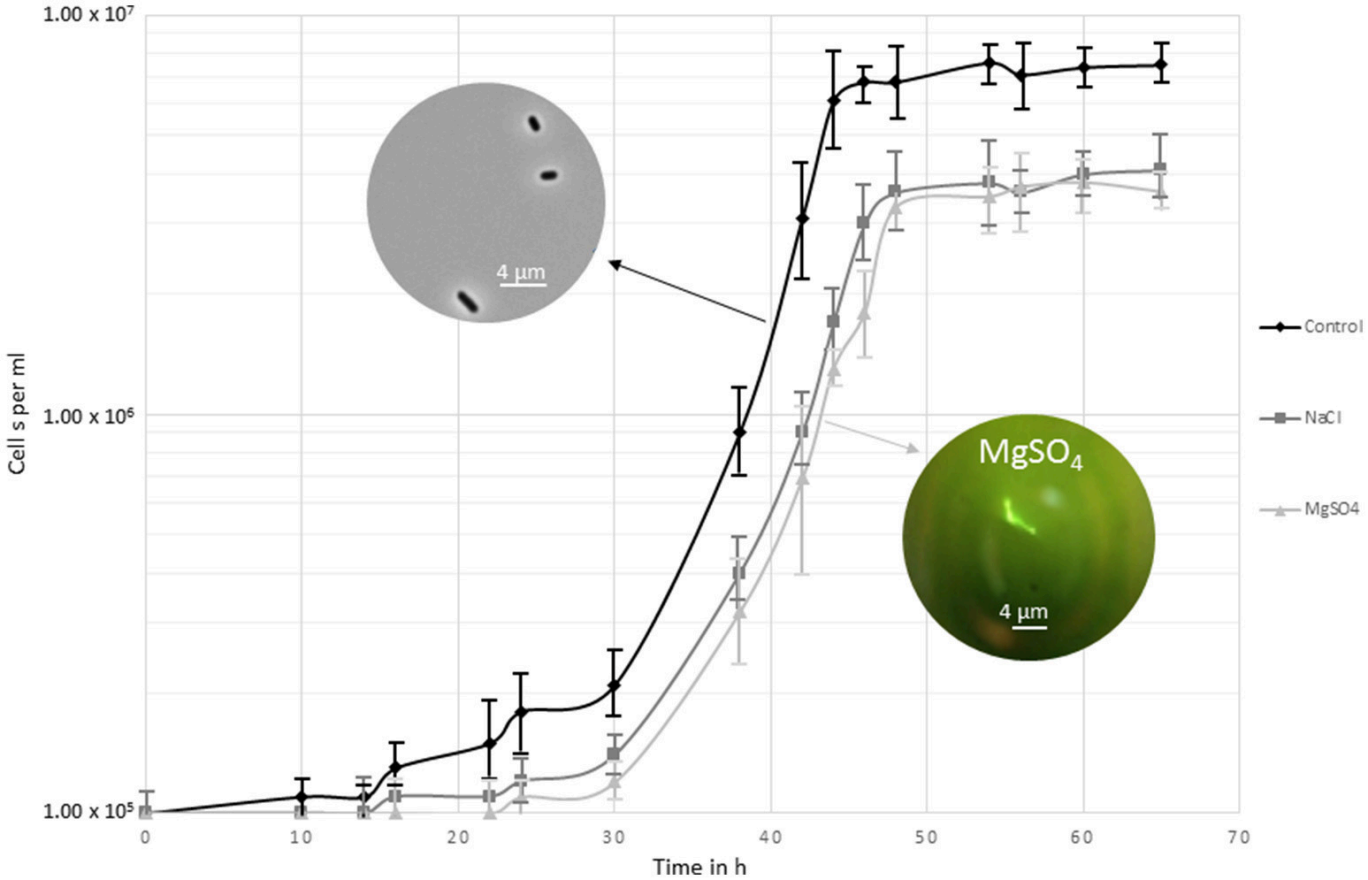
Growth curve of *Y. intermedia* MASE-LG-1 cultivated under the three investigated conditions applied for the metabolome experiments; optimal anoxic conditions (30°C, pH 7, 0 M NaCl, 0 M MgSO_4_), NaCl salt stressed (30°C, pH 7, 0.4 M NaCl) and MgSO_4_ salt stressed (30°C, pH 7, 0.7 M MgSO_4_). Growth was determined by direct cell counting. The images display the morphological change when grown in control conditions and in MgSO_4_.

Growth of *Y. intermedia* strain MASE-LG-1 was investigated under a broad range of MgSO_4_ and NaCl concentrations. Under optimal enrichment conditions, i.e., in MASE I medium without added salt, a maximal cell density of approximately 10^7^ cells per ml was observed (Figure 1). *Yersinia intermedia* MASE-LG-1 was able to grow in the range of 0–1.1 M NaCl and 0–1.5 M MgSO_4_ with an optimal growth at 0 M. However, under increasing salt concentration, the growth rate was lower and a prolonged lag phase was generally observed with increasing salt concentrations. An overall lower maximal cell density was observed in salt-stress conditions compared to the control, which was further decreased with increasing salt concentration. At the highest salt concentrations tested, an increased cell density was only observed after 7 days of incubation which reached a maximum of 3.0 × 10^5^ cells per ml.

We also observed morphological changes in enrichment cultures containing MgSO_4_ (Figure 1). From 0.5 to 1.5 M MgSO_4_, the organism formed chains. This was not observed in medium containing NaCl, indicating a potentially different response of *Y. intermedia* MASE-LG-1 cells to the two salts tested. Higher concentrations 2 and 1.5 M for MgSO_4_ and NaCl respectively, prevented cell growth.

Assessing the physiological adaptations to hyperosmotic conditions requires the profiling of the intracellular metabolite levels under the specific conditions. To be able to monitor a change in the metabolome, we chose conditions where salt stress was induced but allowed cells to multiply to reach a biomass sufficient (approximately 10^7^ cells per ml) for metabolite extraction. However, the doubling rate was slower compared to optimal growth conditions (30°C, pH 7, in the absence of NaCl and MgSO_4_). For salt stress, the highest NaCl concentration to reach sufficient biomass was chosen and a water activity of 0.975 was determined. To exclude the effect of different osmotic pressures, the MgSO_4_ concentration was adjusted to the same water activity which corresponded to 0.7 M. This approach allowed us to make a direct comparison between the two different salts without bias due to different water activity conditions. Cells were harvested after 48 h of incubation once they had reached the stationary phase.

Based on the growth phase the microbial response to osmotic stress can be divided into three phases (Csonka, 1989). The initial reaction is either efflux (hyperosmotic stress) or influx (hypoosmotic stress) of cell water along the osmotic gradient which leads to rapid shrinkage or swelling of the cytoplasm (phase one). To restore the turgor and volume, biochemical re-adjustment are made (phase two). Hyperosmotic conditions, as investigated here, result in increased transport or synthesis of compatible solutes. Finally, growth is resumed under the new conditions maintaining the biochemical adjustments (phase three). In our study, as we allowed growth to occur in the salt conditions, we have examined the metabolome of the cells in phase three.

The extracted metabolites were analysed via hydrophilic interaction liquid chromatography (HILIC) technique. Principal component analysis revealed three distinct clusters containing only samples from each experimental condition. The clear separation between PCA groups indicates that the treated groups differentiate with respect to the control group (Supplementary Figure 1).

### Global metabolome identification of *Y. intermedia* MASE-LG-1 unstressed and stressed with MgSO_4_ and NaCl: differential global metabolic response was observed

**Figure 2:**
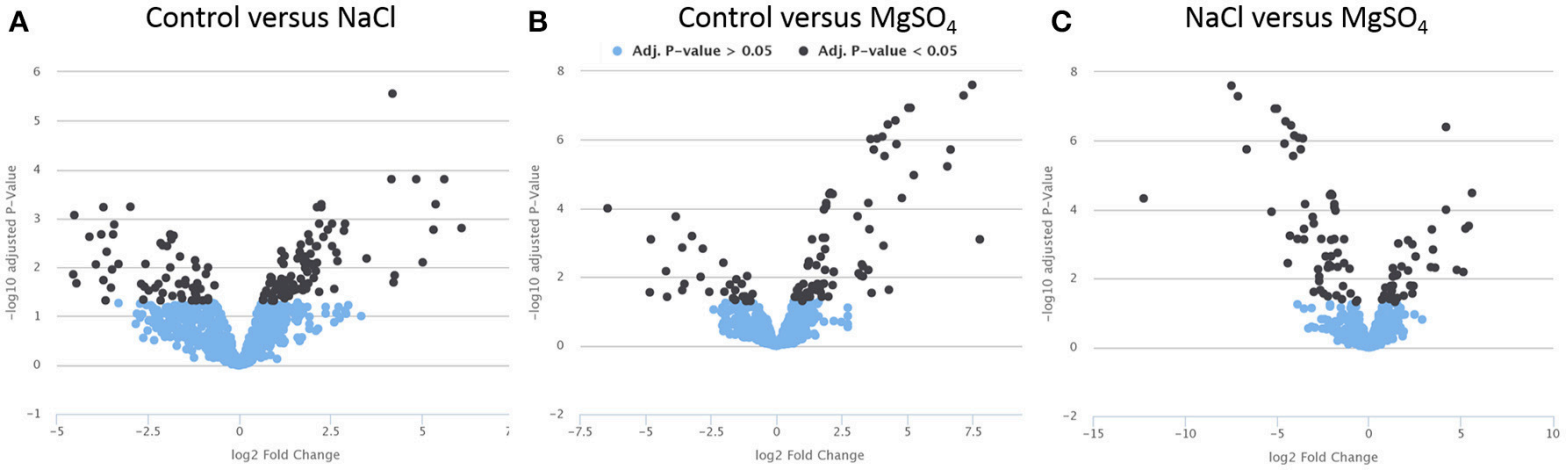
Volcano plots displaying significance (y-axis) vs. fold-change (x-axis). Metabolite data for *Y. intermedia* MASE-LG-1 grown under non-stressed conditions (control, C) and salt-stressed condition with identical water availability in NaCl and MgSO_4_. Pairwise comparison of **(A)** control and NaCl relative to control, **(B)** control and MgSO_4_ relative to control, and **(C)** NaCl and MgSO_4_ relative to NaCl. The plot is generated by plotting the negative log (base 10) of the adjusted *p*-value (<0.05) on the y-axis. Significant and insignificant peaks are represented by dark blue and light blue data points, respectively.

To obtain detailed information about significant relative metabolite changes between control and different salts, the online platform PiMP was used. When putatively assigned, one peak can be represented by more than one metabolite. Therefore, an absolute quantification of the number of metabolites that change is difficult to achieve. A total of 47 unique compounds matched to a known standard. Three group-wise comparisons were undertaken to identify differences (fold changes, see Figure 2). Each fold change has associated significance statistics. Peaks with an adjusted *p*-value less than 0.05 were considered significant. We performed three one group-wise comparisons (e.g., control vs. NaCl, Figure 2A; control vs. MgSO_4_, Figure 2B; and NaCl vs. MgSO_4_, Figure 2C). Annotated metabolites were assigned putatively on the basis of mass.

In the first instance we looked at the significant different effects of each salt individually compared to the control sample. The vast majority of peaks did not change significantly and are therefore considered to represent the metabolites involved in the core metabolism required for the maintenance of cell growth and survival. The peaks changing significantly are considered adaptation to the environmental osmotic stress. For NaCl, a total of 153 peaks (17 identified and 136 annotated) were detected that showed significant different responses in either the salt stressed sample or the control. A total of 94 peaks (most significant changing and identified in both: ornithine, sn-glycero-3-phosphate) showed increased intensity under salt stress compared to 57 peaks (most significant changing and identified in both: L-homoserine, L-threonine) which revealed a higher intensity in the control sample. For MgSO_4_, a total of 108 peaks (5 identified and 103 annotated) were detected that showed significant different responses in either the salt stressed sample or the control. Seventy-three percent of the significantly different metabolites (most significant changing and identified in both: L-asparagine and ornithine) were increased in the MgSO_4_ treated sample. When comparing MgSO_4_ against NaCl, 93 peaks changed significantly. In more detail, 12 identified compounds significantly changed and 83 annotated peaks were detected that showed significant different responses. A total of 54 peaks (most significant changing and identified in both: L-proline, uridine) revealed a significantly increased log2 fold change in the MgSO_4_ sample, compared to 39 (most significant changing and identified in both: choline phosphate, itaconate) peaks in the NaCl samples.

**Figure 3:**
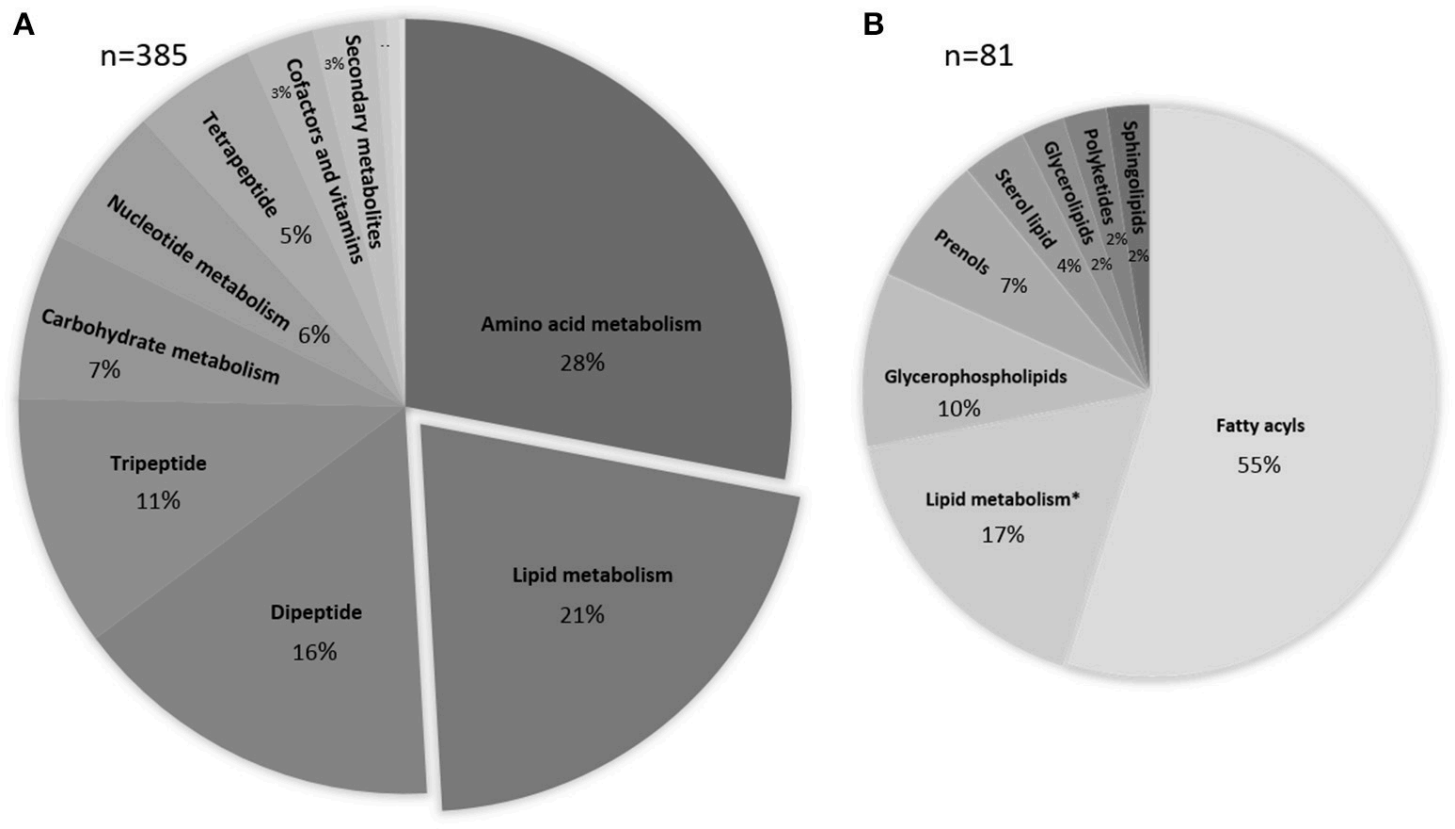
Pie-chart representing the percentage and biological categories of the identified or putatively annotated metabolites based upon their putative functions. Assignment was performed using KEGG database. **(A)** Data are comprised of 385 peaks, the three smallest sections represent energy metabolism, xenobiotics drugs etc., xenobiotics biodegradation and metabolism with <1%. **(B)** Detailed information about the various lipid compounds. * depicts peaks of metabolites that are involved in the lipid metabolism but are grouped within a different chemical class.

The metabolomics data processed with IDEOM were used to obtain an overview of chemical compounds identified and grouped as a function of their role in the microbial metabolism. A total of 557 metabolites were observed, with 168 thereof not being allocated to any metabolic map. The majority of the remaining 385 identified or putatively annotated metabolites are part of the amino acid and lipid metabolism (28 and 21% respectively, Figure 3).

### Main changes were observed in the amino acid metabolism

To identify which parts of the metabolism are affected by the stress and to pinpoint the differences between the salts, the metabolites were mapped to the Kyoto Encyclopedia of Genes and Genomes (KEGG) database against Brite organism *Yersinia intermedia* Y228 (KEGG abbreviation yin631). It is noteworthy that the metabolisms, and therefore the metabolomes, differ from one organism to another. This implies that the detectable number of metabolites referenced in any available database is organism-dependent (Merlet et al., 2016).

A total of 117 maps and metabolic pathways were identified for the KEGG organism yin631. The significantly identified and annotated metabolites mapped to 86 KEGG metabolic maps (Table 1). We observed significant changes of numerous metabolites mainly in the amino acid metabolism and translation as well as membrane transport. No significant changes were observed in the metabolism of terpenoids and polyketids, transcription and replication and repair pathways. The coverage for other pathways was below 25% and only very small changes were detected in the energy metabolism, the glycan biosynthesis and metabolism, metabolisms of cofactors and vitamins and cellular processes like cell motility and quorum sensing pathways.

**Table 1:** KEGG pathway maps for *Y. intermedia* yin631.

		**Kegg identifier**	**C vs. NaCl**	**C vs. MgSO_4_**	**NaCl vs. MgSO_4_**
Metabolism	Global and overview maps	01100 Metabolic pathways	38	20	20
		01110 Biosynthesis of secondary metabolites	27	15	15
		01120 Microbial metabolism in diverse environments	21	12	11
		01130 Biosynthesis of antibiotics	25	14	13
		01200 Carbon metabolism	5	2	4
		01210 2-Oxocarboxylic acid metabolism	10	5	4
		01230 Biosynthesis of amino acids	18	9	9
		01220 Degradation of aromatic compounds	6	2	3
	Carbohydrate metabolism	00010 Glycolysis / Gluconeogenesis	1	1	1
		00020 Citrate cycle (TCA cycle)	1	0	1
		00030 Pentose phosphate pathway	0	1	1
		00040 Pentose and glucuronate interconversions	0	1	0
		00051 Fructose and mannose metabolism	0	1	1
		00052 Galactose metabolism	1	2	1
		00053 Ascorbate and aldarate metabolism	1	2	3
		00500 Starch and sucrose metabolism	0	1	1
		00520 Amino sugar and nucleotide sugar metabolism	1	3	2
		00620 Pyruvate metabolism	1	0	1
		00630 Glyoxylate and dicarboxylate metabolism	5	1	3
		00640 Propanoate metabolism	3	1	2
		00650 Butanoate metabolism	3	0	3
		00660 C5-Branched dibasic acid metabolism	1	2	3
		00562 Inositol phosphate metabolism	0	1	1
	Energy metabolism	00190 Oxidative phosphorylation	1	0	1
		00680 Methane metabolism	2	1	2
		00910 Nitrogen metabolism	2	0	1
		00920 Sulfur metabolism	4	0	3
	Lipid metabolism	00061 Fatty acid biosynthesis	2	2	0
		00071 Fatty acid degradation	0	0	0
		00121 Secondary bile acid biosynthesis	0	0	0
		00561 Glycerolipid metabolism	1	0	0
		00564 Glycerophospholipid metabolism	2	2	3
		00565 Ether lipid metabolism	1	0	0
		00600 Sphingolipid metabolism	1	0	0
		00590 Arachidonic acid metabolism	0	0	0
		00592 alpha-Linolenic acid metabolism	0	0	0
		01040 Biosynthesis of unsaturated fatty acids	1	1	0
	Nucleotide metabolism	00230 Purine metabolism	8	4	4
		00240 Pyrimidine metabolism	3	0	2
	Glycan biosynthesis and metabolism	00540 Lipopolysaccharide biosynthesis	0	0	0
		00550 Peptidoglycan biosynthesis	1	1	0
		00511 Other glycan degradation	1	1	1
	Metabolism of cofactors and vitamins	00730 Thiamine metabolism	1	0	0
		00740 Riboflavin metabolism	0	0	0
		00750 Vitamin B6 metabolism	0	0	0
		00760 Nicotinate and nicotinamide metabolism	3	0	3
		00770 Pantothenate and CoA biosynthesis	1	1	1
		00780 Biotin metabolism	2	1	0
		00785 Lipoic acid metabolism	0	0	0
		00790 Folate biosynthesis	1	0	1
		00670 One carbon pool by folate	0	0	0
		00860 Porphyrin and chlorophyll metabolism	3	0	2
		00130 Ubiquinone and other terpenoid-quinone biosynthesis	1	0	1
	Biosynthesis of other secondary metabolites	00332 Carbapenem biosynthesis	2	0	2
		00261 Monobactam biosynthesis	3	1	2
		00521 Streptomycin biosynthesis	0	2	2
		00525 Acarbose and validamycin biosynthesis	0	0	0
		00401 Novobiocin biosynthesis	2	0	2
	Xenobiotics biodegradation and metabolism	00362 Benzoate degradation	1	0	0
		00627 Aminobenzoate degradation	3	0	2
		00364 Fluorobenzoate degradation	0	0	0
		00625 Chloroalkane and chloroalkene degradation	2	2	2
		00361 Chlorocyclohexane and chlorobenzene degradation	4	0	2
		00623 Toluene degradation	1	0	0
		00633 Nitrotoluene degradation	0	0	1
		00930 Caprolactam degradation	1	1	0
		00626 Naphthalene degradation	1	0	1
Genetic Information Processing	Transcription	03020 RNA polymerase	0	0	0
	Translation	03010 Ribosome	0	0	0
		00970 Aminoacyl-tRNA biosynthesis	9	1	5
	Folding, sorting and degradation	03060 Protein export	0	0	0
		04122 Sulfur relay system	1	0	1
		03018 RNA degradation	0	0	0
Environmental Information Processing	Membrane transport	02010 ABC transporters	9	5	5
		02060 Phosphotransferase system (PTS)	2	2	1
		03070 Bacterial secretion system	5	1	3
	Signal transduction	02020 Two-component system	0	0	0
Cellular Processes	Cellular community - prokaryotes	02024 Quorum sensing	1	0	0
	Cell motility	02030 Bacterial chemotaxis	1	1	1
		02040 Flagellar assembly	0	0	0
Human Diseases	Drug resistance: Antimicrobial	01501 beta-Lactam resistance	0	0	0
		01502 Vancomycin resistance	2	1	0
		01503 Cationic antimicrobial peptide (CAMP) resistance	0	0	0

*The numbers indicate the number of significantly changing peaks identified for each map or pathway*.

**Figure 4:**
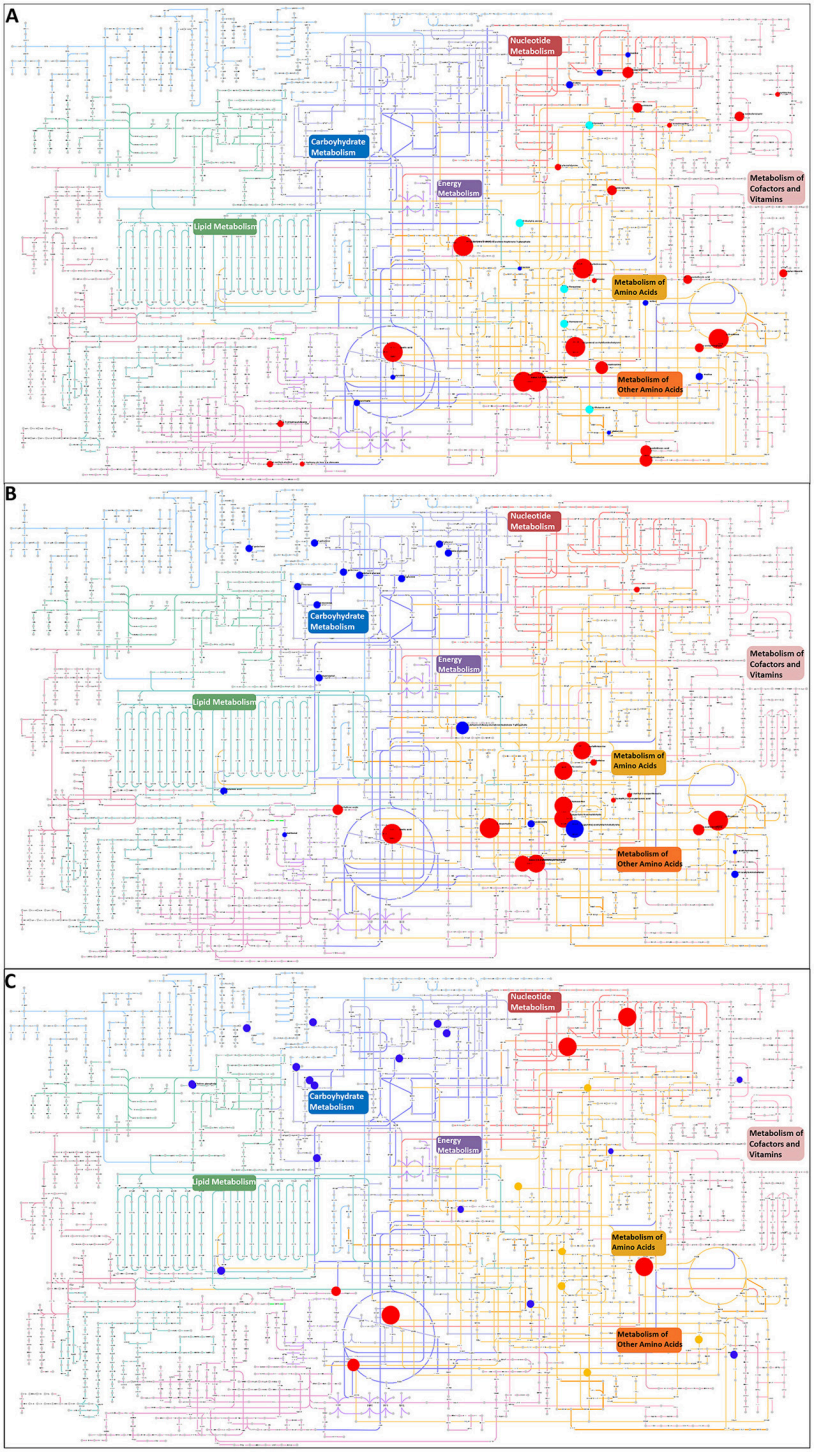
Visualization of metabolites that are significantly changed (adjusted *p*-value < 0.05) in the salt-stressed sample NaCl on the KEGG metabolic pathway map using Pathway Projector (Kono et al., 2009). Color code for pathway categories: aqua represents glycan biosynthesis and metabolism, blue represents carbohydrate metabolism, green represents lipid metabolism, red represents nucleotide metabolism, purple represents energy metabolism, yellow represents amino acid metabolism, pink represents metabolism of cofactors and vitamins, dark red represents biosynthesis of secondary metabolites, orange represents metabolism of other amino acids, and magenta represents biodegradation and metabolism of xenobiotics. Blue circles indicate a decrease and red circles an increase in the salt-stressed sample compared to the control. Orange circles depict metabolites only found in the salt stress sample, whereas cyan circles depict metabolites only found in the control sample. Size indicates the relative increase but is cut off at 3 due to overlay issues. Pairwise comparison of **(A)** control and NaCl relative to control, **(B)** control and MgSO_4_ relative to control, and **(C)** NaCl and MgSO_4_ relative to NaCl.

All metabolites that were putatively identified for the significantly changing peaks were searched against the KEGG atlas and visualized via Pathway Projector to obtain a systematic overview and to locate the differences (Kono et al., 2009, Figure 4). Some of the metabolites were depicted in areas of pathways without connection to each other. The distribution of the various metabolites indicated a differential response. While we observed clusters in the carbohydrate metabolism under MgSO_4_ stress (Figure 4B) which was absent in the NaCl stressed sample (Figure 4A), we observed a cluster of significantly changing metabolites within the metabolism of amino acids and other amino acids (according to KEGG which represents pathways of non-essential amino acids). In the case of NaCl the majority of them correlated positively with the salt stress. The energy metabolism and redox cofactor regeneration including the glycolysis as well as the tricarboxylic acid cycle and the pentose phosphate pathway were barely affected.

The greatest changes have been observed in the amino acid metabolisms and therefore we mapped the metabolites against the KEGG organism yin631 specific amino acid KEGG map.

**Figure 5:**
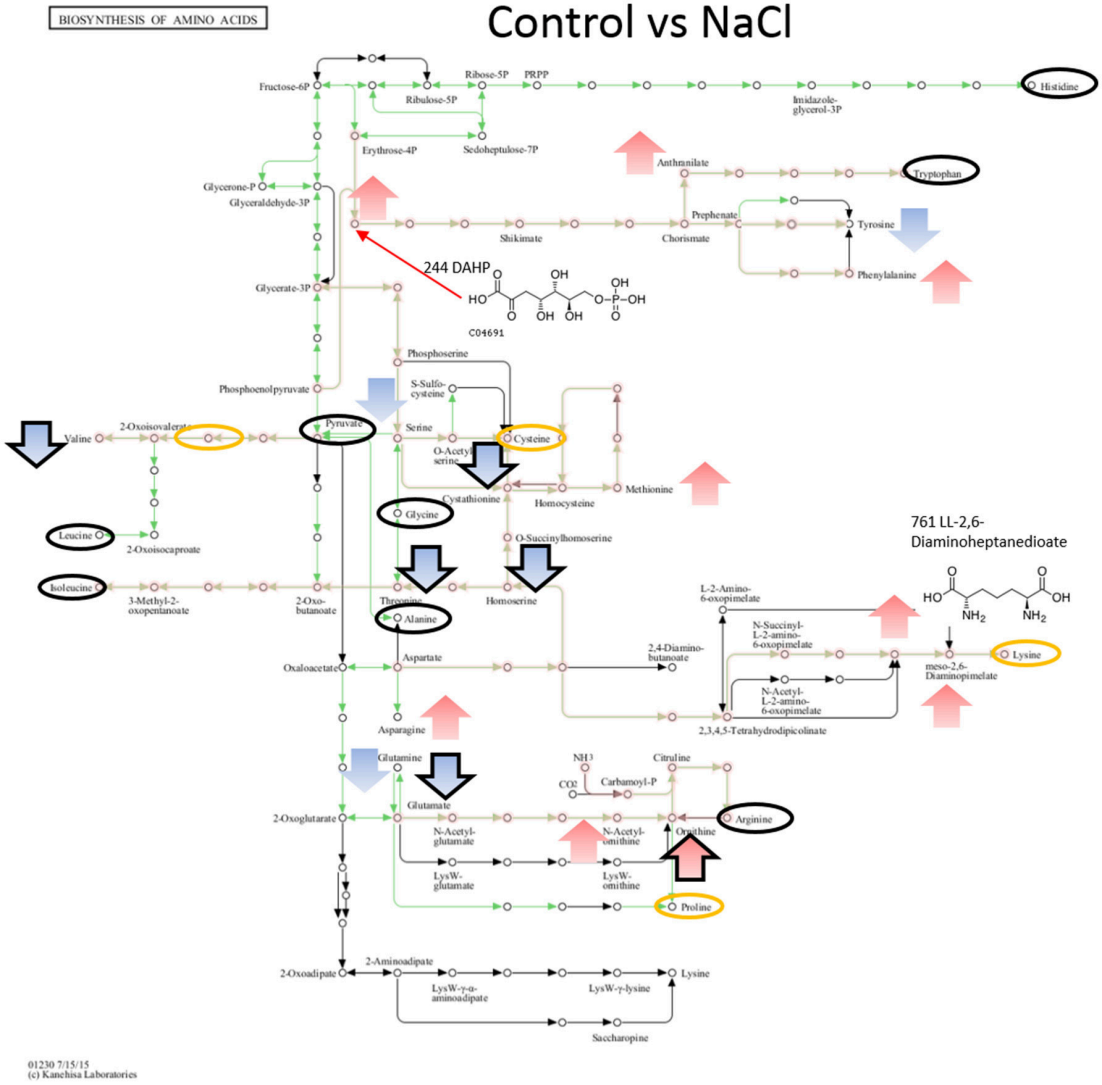
Visualization of metabolites that are significantly changed (adjusted *p*-value < 0.05) in salt stressed sample NaCl on the KEGG global map: Biosynthesis of amino acids 01230. Green lines indicate pathways identified for yin631. Orange lines indicate pathways where metabolites have been identified. Yellow circle: putatively annotated metabolite but not significantly changed. Black circle: identified metabolite, i.e., the peak matches a standard, but is not significantly changed. Arrows indicate increase (red) or decrease (blue) in salt stress samples. Black outline of the arrows depicts an identified metabolite, while no outline indicates a putatively annotated metabolite.

**Figure 6:**
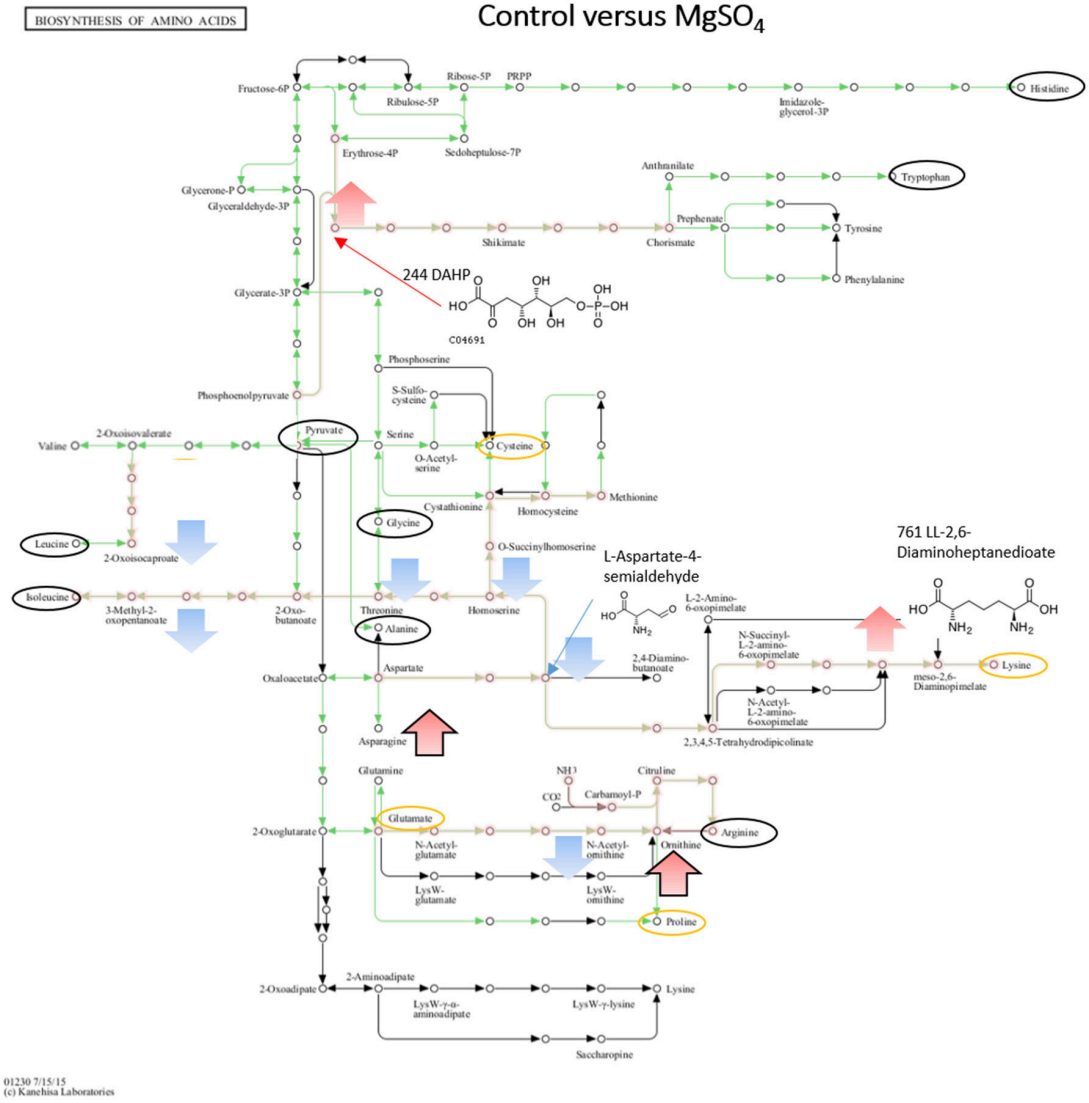
Visualization of metabolites that are significantly changed (adjusted *p*-value < 0.05) in salt stressed sample MgSO_4_ on the KEGG global map: Biosynthesis of amino acids 01230. Green lines indicate pathways identified for yin631. Orange lines indicate pathways where metabolites have been identified. Yellow circle: putatively annotated metabolite but not significantly changed. Black circle: identified metabolite, i.e. the peak matches a standard, but is not significantly changed. Arrows indicate increase (red) or decrease (blue) in salt stress samples. Black outline of the arrows depicts an identified metabolite, while no outline indicates a putatively annotated metabolite.

**Figure 7:**
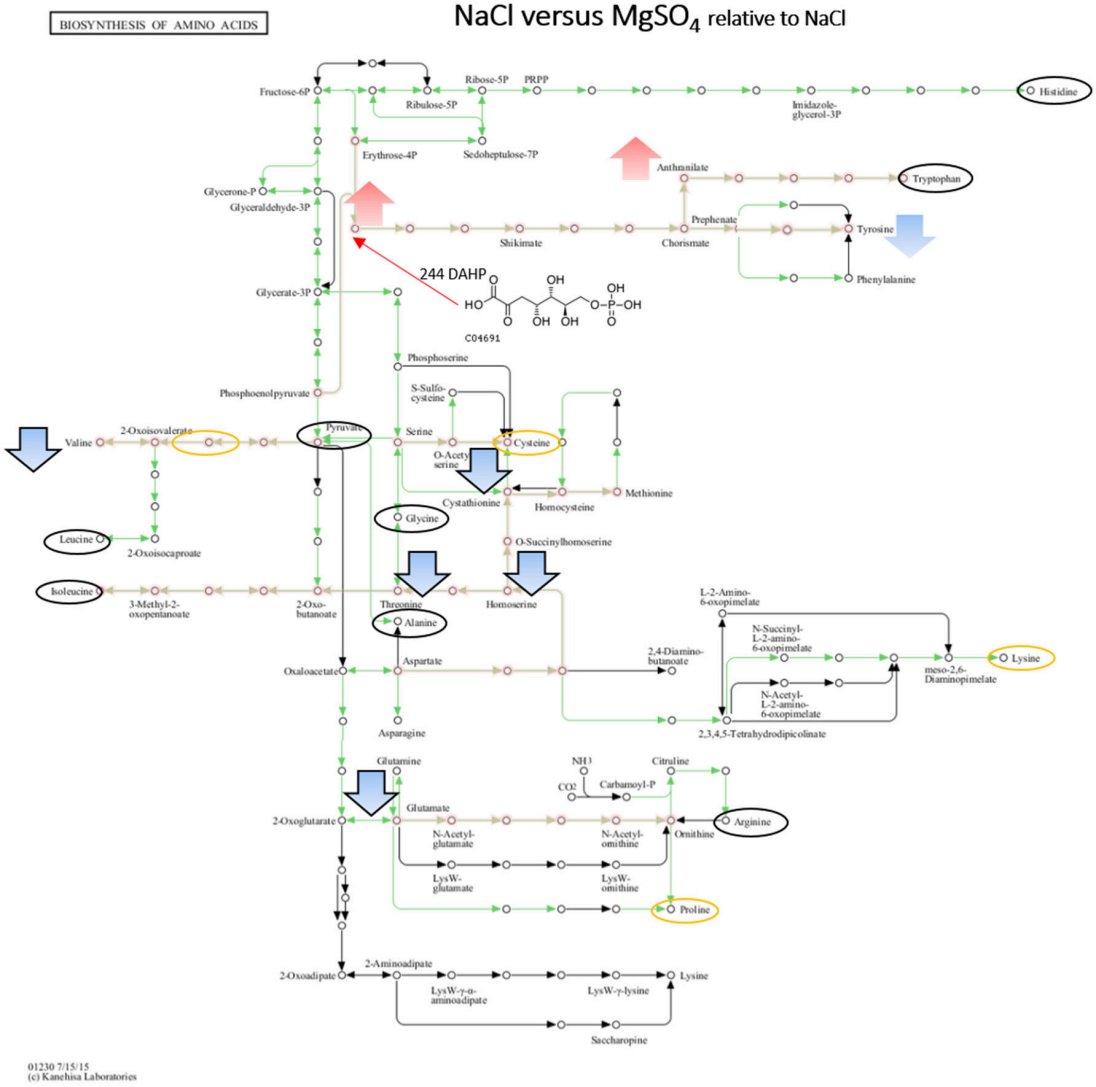
Visualization of metabolites that are significantly changed (adjusted *p*-value < 0.05) under both salts relative to NaCl on the KEGG global map: Biosynthesis of amino acids 01230. Green lines indicate pathways identified for yin631. Orange lines indicate pathways where metabolites have been identified. Yellow circle: putatively annotated metabolite but not significantly changed. Black circle: identified metabolite, i.e., the peak matches a standard, but is not significantly changed. Arrows indicate increase (red) or decrease (blue) in MgSO_4_ stressed samples. Black outline of the arrows depicts an identified metabolite, while no outline indicates a putatively annotated metabolite.

When comparing NaCl and MgSO_4_ stressed samples against the control, seven metabolites (tyrosine, valine, threonine, homoserine, O-acetylserine, serine) were significantly decreased while 10 (asparagine, methionine, glutamine, glutamate, meso-2,6-diaminopimelate, N-acetyl-ornithine, ornithine, anthranilate, DAHP, phenylalanine, LL-2,6-diaminoheptane-diole) were significantly increased in the former (Figure 5). Six metabolites (2-osoiscaproate, 3-Methyl-2-oxopentanoate, threonine, homoserine, L-asparate-4-semialdehyde, N-acetyl-ornithine) were significantly decreased while asparagine, ornithine, LL-2,6-diaminoheptanedioate, and DAHP were significantly increased in the latter (Figure 6). Differences between the two salts were detected in eight metabolites (see Figure 7).

### Identification of potentially osmoregulatory signals

**Figure 8:**
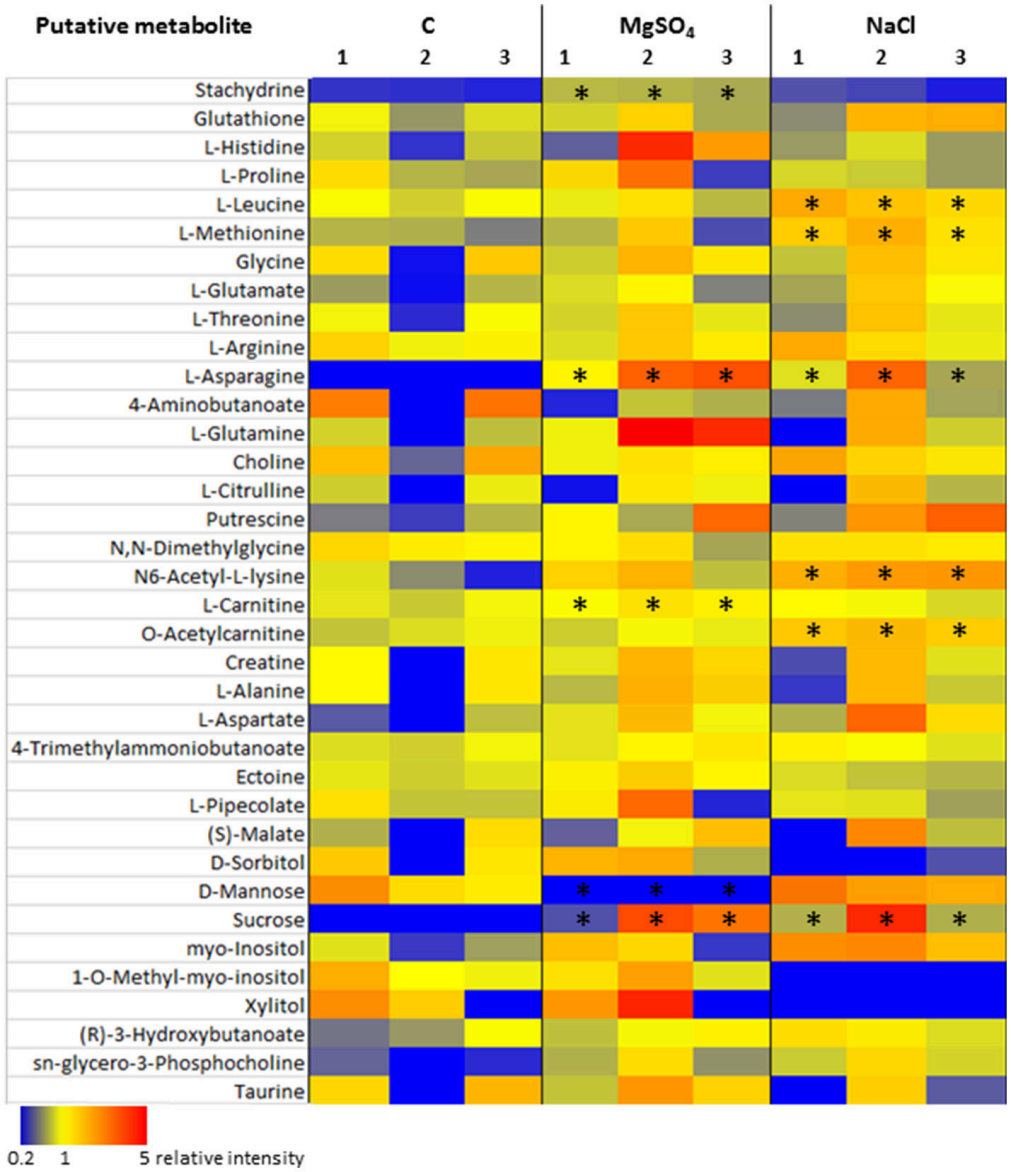
Heatmap of detected putative osmolytes. The individual intensity values (*n* = 3, the numbers at the top of each column indicate the replica) of each condition, e.g., control, NaCl, and MgSO_4_ are shown. Metabolite levels are coloured according to relative intensity (blue = low, red = high). Asterisks mark significant different results.

A detailed insight into one potential metabolic adaptation of *Y. intermedia* MASE-LG-1 to salt stress was achieved by systematically identifying already known osmoprotectants summarized and publicly available in the DEOP database from the overall detected 557 metabolites (Bougouffa et al., 2014). In total, 37 metabolites were identified which are linked to putative osmoprotective activity (see Table 2). Thereof, 26 are amino acids, seven belong to carbohydrates and three belong to lipids. Stachydrine was not classified. For the vast majority of these metabolites, only minor changes (relative increase <2) of the salt stressed samples compared to the control were observed (see Figure 8). However, the relative intensity of the amino acid L-asparagine was enhanced 25-fold for MgSO_4_ and 15-fold for NaCl compared to the control. The values for the carbohydrate sucrose were 19- and 17-fold higher in salt-stressed samples compared to the control, respectively.

**Table 2:** List of identified osmolytes.

**Putative metabolite**	**Formula**	**Relative intensity**		**Metabolism**	**Pathway**
		**MgSO_4_**	**NaCl**		
L-Asparagine	C_4_H_8_N_2_O_3_	**25.65**	**15.24**	amino acid	Alanine and aspartate metabolism; Tetracycline biosynthesis; Cyanoamino acid metabolism; Nitrogen metabolism
Glutathione	C_10_H_17_N_3_O_6_S	1.19	1.66	amino acid	Glutamate metabolism; Cysteine metabolism; Glutathione metabolism
L-Histidine	C_6_H_9_N_3_O_2_	3.49	1.08	amino acid	Histidine metabolism; beta-Alanine metabolism
L-Proline	C_5_H_9_NO_2_	1.63	0.84	amino acid	Arginine and proline metabolism; Novobiocin biosynthesis
L-Leucine	C_6_H_13_NO_2_	1.01	**1.61**	amino acid	Valine, leucine and isoleucine degradation; Valine, leucine and isoleucine biosynthesis
L-Methionine	C_5_H_11_NO_2_S	1.39	**2.46**	amino acid	Methionine metabolism
Glycine	C_2_H_5_NO_2_	1.22	1.17	amino acid	Bile acid biosynthesis; Purine metabolism; Glycine, serine and threonine metabolism; Lysine degradation; Cyanoamino acid metabolism; Glutathione metabolism; Methane metabolism; Thiamine metabolism; Porphyrin and chlorophyll metabolism; Nitrogen metabolism
L-Glutamate	C_5_H_9_NO_4_	1.65	2.10	amino acid	Arginine and proline metabolism; Glutamate metabolism; Histidine metabolism; D-Glutamine and D-glutamate metabolism; Glutathione metabolism; Butanoate metabolism; C5-Branched dibasic acid metabolism; Porphyrin and chlorophyll metabolism; Nitrogen metabolism
L-Threonine	C_4_H_9_NO_3_	1.40	1.29	amino acid	Glycine, serine and threonine metabolism; Valine, leucine and isoleucine biosynthesis; Porphyrin and chlorophyll metabolism
L-Arginine	C_6_H_1_4N_4_O_2_	1.0	1.15	amino acid	Arginine and proline metabolism; Clavulanic acid biosynthesis; D-Arginine and D-ornithine metabolism
4-Aminobutanoate	C_4_H_9_NO_2_	0.36	0.24	amino acid	Arginine and proline metabolism; Glutamate metabolism; beta-Alanine metabolism; Butanoate metabolism
L-Glutamine	C_5_H_10_N_2_O_3_	**6.48**	1.64	amino acid	Glutamate metabolism; Purine metabolism; Pyrimidine metabolism; D-Glutamine and D-glutamate metabolism; Nitrogen metabolism
Choline	C_5_H_13_NO	0.89	1.17	amino acid	Glycine, serine and threonine metabolism; Glycerophospholipid metabolism
L-Citrulline	C_6_H_13_N_3_O_3_	1.36	1.36	amino acid	Arginine and proline metabolism
Putrescine	C_4_H_12_N_2_	2.65	3.53	amino acid	Arginine and proline metabolism; Glutathione metabolism; Alkaloid biosynthesis II
N,N-Dimethylglycine	C_4_H_9_NO_2_	0.83	1.02	amino acid	Glycine, serine and threonine metabolism
N6-Acetyl-L-lysine	C_8_H_16_N_2_O_3_	2.30	**3.42**	amino acid	Lysine degradation
L-Carnitine	C_7_H_15_NO_3_	**1.31**	1.10	amino acid	Lysine degradation
O-Acetylcarnitine	C_9_H_17_NO_4_	1.07	**1.77**	amino acid	Alanine and aspartate metabolism
Creatine	C_4_H_9_N_3_O_2_	1.57	1.14	amino acid	Glycine, serine and threonine metabolism; Arginine and proline metabolism
L-Alanine	C_3_H_7_NO_2_	1.54	1.08	amino acid	Alanine and aspartate metabolism; Cysteine metabolism; Taurine and hypotaurine metabolism; Selenoamino acid metabolism; D-Alanine metabolism; Carbon fixation; Reductive carboxylate cycle (CO_2_ fixation)
L-Aspartate	C_4_H_7_NO_4_	2.99	4.06	amino acid	Alanine and aspartate metabolism; Arginine and proline metabolism; Glycine, serine and threonine metabolism; Lysine biosynthesis; Arginine and proline metabolism; Histidine metabolism; beta-Alanine metabolism; Cyanoamino acid metabolism; Carbon fixation
4-Trimethyl-ammoniobutanoate	C_7_H_15_NO_2_	0.72	**1.44**	amino acid	Lysine degradation
Ectoine	C_6_H_10_N_2_O_2_	1.43	0.87	amino acid	Glycine, serine and threonine metabolism
L-Pipecolate	C_6_H_11_NO_2_	1.51	0.87	amino acid	Lysine degradation; Alkaloid biosynthesis II
Betaine	C_5_H_11_NO_2_	**1.81**	**1.56**	amino acid	Glycine, serine and threonine metabolism
(S)-Malate	C_4_H_6_O_5_	1.47	1.54	carbohydrate	Citrate cycle; Glutamate metabolism; Alanine and aspartate metabolism; Pyruvate metabolism; Glyoxylate and dicarboxylate metabolism; Carbon fixation; Reductive carboxylate cycle (CO_2_ fixation)
Sucrose	C_12_H_22_O_11_	**19.02**	**16.65**	carbohydrate	Galactose metabolism; Starch and sucrose metabolism
D-Sorbitol	C_6_H_14_O_6_	1.43	0.12	carbohydrate	Fructose and mannose metabolism; Galactose metabolism
D-Mannose	C_6_H_12_O_6_	**0.08**	1.33	carbohydrate	Fructose and mannose metabolism; Galactose metabolism
myo-Inositol	C_6_H_12_O_6_	1.81	**3.35**	carbohydrate	Inositol metabolism; Galactose metabolism; Ascorbate and aldarate metabolism; Streptomycin biosynthesis; Inositol phosphate metabolism
1-O-Methyl-myo-inositol	C_7_H_14_O_6_	1.07	0.00	carbohydrate	Inositol phosphate metabolism
Xylitol	C_5_H_12_O_5_	1.71	0.00	carbohydrate	Pentose and glucuronate interconversions
(R)-3-Hydroxybutanoate	C_4_H_8_O_3_	1.41	1.63	lipid	Synthesis and degradation of ketone bodies; Butanoate metabolism
sn-glycero-3-Phosphocholine	C_8_H_2_0NO_6_P	2.93	3.44	lipid	Glycerophospholipid metabolism; Ether lipid metabolism
Taurine	C_2_H_7_NO_3_S	1.37	0.60	lipid	Bile acid biosynthesis; Taurine and hypotaurine metabolism
Stachydrine	C_7_H_13_NO_2_	**2.32**	**1.11**	0	Stachydrine degradation

*Metabolite levels for each experimental group are shown. Levels are expressed as mean peak intensity relative to the mean peak intensity of the control group. Numbers indicated in bold indicates statistically significance (adjusted p-value < 0.05)*.

Similarities and differences were observed in other amino acids in both salt treatments. An increase (relative intensity >2) was observed in L-histidine, L-glutamine, putrescine, N6-acetyl-L-lysine, L-aspartate, sn-glycero-3-phosphocholine, and stachydrine in MgSO_4_ samples whereas a decrease (relative intensity <0.5) in 4-aminobutanoate and D-mannose was observed. While the same trend was observed in NaCl treated samples for putrescine, N6-acetyl-L-lysine, L-aspartate, sn-glycero-3-phosphocholine and 4-aminobutanoate, the following metabolites L-methionine, L-glutamate and N6-acetyl-L-lysine were increased, whereas 1-O-methyl-myo-Inositol and xylitol were absent.

### Changes in lipids

It is known that bacteria can alter the lipid composition when experiencing osmotic stress (Nichols et al., 2000; Romantsov et al., 2009; Tsuzuki et al., 2011). More specifically, lipid packing, fatty acid saturation and phospholipid composition are affected. Therefore, we screened for changes within the relative intensity of the identified and putatively identified lipids in our metabolomes. In total, 21% of the metabolites were grouped within the lipids with the majority belonging to the fatty acyls and glycerophospholipids (Figure 3B). The metabolites that were not classified as lipids but are involved in the lipid metabolism and were grouped within lipid metabolism are mainly involved in fatty acid biosynthesis and glycerophospholipid metabolism (for a complete list, see Supplementary Table 1). A heatmap was generated to detect relative changes in the salt stressed samples compared to the control sample. We observed a decrease (relative intensity <0.7) under salt stress conditions in 69% of the lipids while only nine lipids revealed an increase in intensity and one third remained relatively unchanged (relative intensity range >0.7–1.3). No significant differences were observed based on the different salts. The glycerophospholipids revealed a negative trend, while the fatty acyls showed a mixed response with a tendency to decrease. The observed depletion is not correlated with the saturation state of the lipids. The polyketide putatively annotated as Spinochalcone C showed a significant increase. We observed an increase of 2-C-methyl-D-erythritol 4-phosphate in both salt stressed samples.

## Discussion

*Yersinia intermedia* MASE-LG-1, affiliated with the family Enterobacteriaceae, is a Gram-negative, facultatively anaerobic, motile, rod-shaped bacterium (Brenner et al., 1980; Cockell et al., 2017). Members of the genus *Yersinia* are ecologically diverse. *Y. intermedia* has been found in freshwater and marine environments (Shayegani et al., 1981), mammals (Babujee et al., 2013) and Mars analog environments (Cockell et al., 2017), and although it is not considered a human-pathogen, traces were found in urine, stool and wounds (Butler et al., 1984; Farmer et al., 1985; Punsalang et al., 1987). Survivability tests have shown that it can withstand diverse individual and combined stresses like desiccation, X-rays and osmotic stress (Beblo-Vranesevic et al., 2017b). *Yersinia intermedia* MASE-LG-1 must have evolved metabolic adaptations to counteract the experienced stress and to minimize damage caused by different stressors. Thus it is of interest to identify how it can adjust to potentially diverse ionic conditions that might be found in these environments.

In this study, we used an untargeted high-throughput approach allowing us to look at the global trends in the metabolic response of *Y. intermedia*. Although this approach cannot identify all metabolites (Villas-Bôas et al., 2007) it allows us to investigate a large number of functional categories that are subject to change. We observed changes in carbohydrates, amino acids, peptides, lipids to cofactors and vitamins, showing that the salts alter the global cellular metabolism. It is important to mention that the measured changes might be specific with regard to the culture conditions such as temperature, growth phase, salt concentrations as well as nutrient availability for uptake of metabolites. Therefore, the results represent a screenshot at the tested condition.

### Similar responses were observed in putative osmolytes and lipid changes in NaCl and MgSO_4_

Some changes in the metabolome were similar in both, NaCl and MgSO_4_. In both salts, certain potential osmolytes were upregulated, in particular sucrose and L-asparagine revealed the highest changes.

Only a few carbohydrates are used by bacteria for osmotic balance due to their reactivity with proteins (Roberts, 2005). However, sucrose, a non-reducing disaccharide, is an effective osmoprotectant in some halotolerant and halophilic organisms (Roberts, 2005). Sucrose is rarely synthesized although it has been shown for cyanobacteria, sulfur bacteria and proteobacteria (Reed et al., 1986; Welsh and Herbert, 1993; Lunn et al., 1999). Screening the genome of yersiniae (unpublished data) revealed that this bacterium does not possess a gene for sucrose synthase. However, the search revealed a gene for a sucrose porin which indicates that the cells do not synthesize but rather take up sucrose from the environment. Apart from the observed accumulation in *Y. intermedia* MASE-LG-1, sucrose was described to be critical for stationary phase survival under salt stress conditions in *Synechosystis* sp. It was hypothesized to regulate metabolic pathways that are active under the nutritional stress conditions of stationary phase (Deplats et al., 2005). In addition to its ability to counteract osmotic stress, *in vitro* studies have shown that sucrose and other sugars are able to stabilize biomolecules and membrane lipids (Welsh, 2000). Apart from being a powerful osmoprotectant, it has been shown that sucrose was used as a carbon and energy source (Gouffi et al., 1999), which might also be the case for *Y. intermedia* MASE-LG-1. API tests have shown that *Y. intermedia* MASE-LG-1 can utilize sucrose (Beblo-Vranesevic et al., 2017b). The KEGG database also revealed three invertase-like enzymes (EC:3.2.1.26) allowing to break down sucrose into glucose and fructose.

L-asparagine is known to be an essential intermediate in bacterial metabolism providing nitrogen for growth. With regard to its effect as an osmoprotectant in bacteria little is known whereas there have been reports of plants that used L-asparagine suggesting that asparagine functions as a compatible solute under conditions of stress (Imamul Huq and Larher, 1983; Lehle et al., 1992). L-asparagine is a substrate of transferases and is implicated in the trigger reaction initiating peptide and protein synthesis. In addition, L-asparagine also serves as a transport and storage substance for the component parts of many amino acids (Waelsch, 1952). *Escherichia coli* actively accumulates asparagine endogenously up to several-fold above the apparent biosynthetic pool of the amino acid and 100-fold above the external medium for protein synthesis (Willis and Woolfolk, 1975). *Mycobacterium tuberculosis* accumulates L-asparagine under acid stress but is also used for nitrogen assimilation into glutamine and glutamate (Gouzy et al., 2014). In *Y. intermedia* MASE-LG-1 L-asparagine might have similar functions.

Apart from L-asparagine and sucrose, we identified additional potential compatible solutes that were also accumulated in both salts. The results showed an increase of the two metabolites glutamate (2-fold increase in MgSO_4_ and NaCl compared to the control) and glutamine (6-fold and 2-fold increase for MgSO_4_ and NaCl, respectively). Glutamate is an important molecule playing a role in various metabolic processes such as protein synthesis, glycolysis, gluconeogenesis, the citric acid cycle and the urea cycle (Berg et al., 2007). It is the main source of nitrogen for the production of N-amine and involved in transamination reactions at the core of the amino acid metabolism. In addition, it is known from various studies that the cytoplasmic levels of glutamate increase in most prokaryotes under induced high osmotic stress (Dinnbier et al., 1988). Despite a described 10-fold increase of glutamate level which accounts for approximately 90% of free amino acids to osmotic stress in Gram-negative bacteria (Tempest et al., 1970), it is not considered to have a great effect as a compatible solute. In addition, the level is dependent on the growth phase and the osmolarity of the growth medium (Richey et al., 1987). Consequently, in *Yersinia intermedia* MASE-LG-1 it is not likely to be the main factor that drives the maintenance of cytoplasmic osmolarity but rather play an important role within the urea cycle in order to regulate and maintain the physiological pH level (Hu et al., 2010). Enteric bacteria assimilate ammonia into glutamine which is a precursor of glutamate. These two metabolites provide a nitrogen source for other biomolecules (Reitzer, 2003). Therefore, the higher levels during the stress response might be explained as a consequence of the increased need for nitrogen containing metabolites due to their function as key metabolites linking nitrogen and carbon metabolism and are directed from the pentose phosphate pathway and citric acid cycle (Gottschalk, 1985; Berg et al., 2007; Joghee and Jayaraman, 2014).

We observed a significant increase (2-fold) of glycine betaine in both salt stressed samples compared to the control sample. Glycine betaine, a widely distributed and effective osmoprotectant in bacteria (Sutherland et al., 1986), is also potent for *Yersinia enterocolitica* and other members of the family Enterobacteriaceae (Le Rudulier and Bouillard, 1983). For *Y. enterocoliticia* a 40-fold increase was observed. While cyanobacteria and some other CO_2_-fixing prokaryotes are able to carry out *de novo* synthesis of glycine betaine, most other bacteria including *Y. enterocolitica* have a transport system in place (Park et al., 1995). It is known that enteric bacteria do not use glycine betaine as a carbon or nitrogen source (Le Rudulier and Bouillard, 1983); thus, glycine betaine might be used for other housekeeping reactions in *Y. intermedia* MASE-LG-1.

We hypothesize for *Y. intermedia* MASE-LG-1 that many of the detected osmolytes have a small effect as potential osmoprotectants, as reflected in the low magnitude of the metabolite concentration changes. While L-asparagine and sucrose may be the major species contributing to osmoprotection, it is the sum of several solutes at moderate concentration which might counteract the osmotic stress. Holistically, they have the potential to contribute to a cumulative osmotic response in addition to the established major compatible solutes. A similar effect was reported for *E. coli, Rhodobacter sphaeroides*, and *Paracoccus versutus* (Record et al., 1998; Sévin et al., 2016). The composition of the compatible solutes can be altered based on growth phase as well as carbon and nitrogen availability. Furthermore, the specific osmoadaptive strategies might also vary depending on the nutrient and compatible solute availability of the habitat and therefore the results reflect only one potential response.

Apart from their osmoprotective potential, these metabolites may have secondary benefits. They can act as general metabolites being involved in protection of cells against other environmental stresses prevalent in the organism's habitat (Welsh, 2000). They can contribute to protein stabilization or interact with cytoplasmic membrane lipids (Sévin et al., 2016). One example relevant for *Yersinia*, surviving in permafrost (Cockell et al., 2017), is the increase in resistance to freezing by accumulation of compatible solutes, specifically sugars and polyols (Barbour and Priest, 1986). The mechanisms to counteract stresses such as freezing, desiccation and osmotic stress are similar and the accumulation of compatible solutes protect enzymes from the denaturating effects. It has been shown that the desiccation tolerance can correlate with the accumulation of sucrose (Potts, 1994). Indeed, we observed a high tolerance to desiccation for *Y. intermedia* MASE-LG-1. Beblo-Vranesevic et al. (2017b) documented a decline of survivability by only four orders of magnitude during a storage time of 85 days.

In addition to common changes in osmolytes, we observed similar lipid changes in both NaCl and MgSO_4_-stressed cells. Cells are known to change their lipid composition in salts to counteract changes in turgor pressure (Tsuzuki et al., 2011) for example by changing fatty acid saturation and the composition of phospholipids (Romantsov et al., 2009; Parsons and Rock, 2013). It has been shown that salinity and the proportion of saturated fatty acids correlates positively (Nichols et al., 2000). Our results for both salts did not reflect this finding despite observing a general decreasing trend in the lipids. The lack of correlation suggests that, under our conditions, individual lipids are not crucial to cell function. Permeability and stability can also be altered through cardiolipin and isoprenoid accumulation (Romantsov et al., 2009; Sévin and Sauer, 2014). In both salts, we observed an accumulation in only two of five identified metabolites involved in the isoprenoid pathways. Choline, used by some bacteria as a precursor for phosphatidylcholine, decreased in the salt stressed samples. Choline might have been used to generate phosphatidylcholine which can be found in significant amounts in membranes of more than 10% of all bacteria (Sohlenkamp et al., 2003). While the present knowledge of the fatty acid composition and information about the lipid metabolism is incomplete and due to the extraction method some of the lipids involved may not have been detected such as phosphatidylcholine, the analysis suggests that there have been similar changes in both salts in the overall lipid metabolism. The decrease in lipids suggests that these molecules may have been used either in other housekeeping and maintenance pathways or their biosynthesis was reduced to re-direct their resources toward pathways and processes that are involved in the salt stress response. However, to clarify this a more lipid-targeted approach is required. We did observe an increase in some lipids. The polyketide putatively annotated as Spinochalcone C showed a significant increase. Since the annotation is based on a low confidence level no further interpretation of its role within the metabolism can be made. We observed an increase of 2-C-methyl-D-erythritol 4-phosphate in both salt stressed samples, a compound involved in the isopentenyl pyrophosphate and dimethylallyl pyrophosphate production pathway. These in turn are required for the biosynthesis of molecules used in a diversity of cellular processes such as protein prenylation, cell membrane maintenance, hormone synthesis, protein anchoring and N-glycosylation.

In addition, certain lipid classes are not only structural components but also can be signalling compounds such as sphingolipids (An et al., 2011; Parsons and Rock, 2013). This is characterized by a high turnover rate of lipid species. Sitting at the membrane interface between cells and the environment, sphingolipids convey diverse signal transduction between lipid and lipids, protein and lipid and between protein and protein and stress response pathways. Cholesterol and sphingolipids, both highly flexible and exhibiting a dynamic nature allowing a timely transmission of signal, are required to manage DNA damage and increase tolerance to oxidative and heat-shock stress in *Bacteroides fragilis* (An et al., 2011). The observed decrease in sphingolipids might be associated with osmotic stress response in *Y. intermedia* MASE-LG-1.

### Similarities and differences were observed in the energy pathways and amino acid metabolism

Since compatible solutes can be accumulated to very high intracellular concentrations, they can represent a significant proportion of the total cell carbon and/or nitrogen (Welsh, 2000). This is especially true for organisms which are able to accumulate mixtures of both carbohydrate and nitrogen containing metabolites as seen for *Y. intermedia* MASE-LG-1. While the global metabolism still needs to be maintained at a certain level, the accumulation of L-asparagine and sucrose requires a major energy investment and carbon resources. Therefore, it is likely that other pathways in the global metabolism, such as the urea cycle, are altered while the osmolyte-producing reactions are favoured. Consequently, these down-stream effects may be reflected in a global metabolic change which we have noticed in the differential metabolic response to the different salts. Pairwise comparisons of our samples clearly show that there is a significant differential salt-dependent stress response. A higher number of metabolites were significantly affected in the NaCl stressed sample compared to the MgSO_4_ stressed sample. These differences might be explained by the fact that we have observed a different growth rate and changes in cell shape when growing in MgSO_4_.

In addition, lower metabolic rates in glycolysis, tricarboxylic acid (TCA) cycle, branched-chain amino acid metabolisms and heme biosynthesis have been described being associated with either NaCl salt stress or on the growth phase (Sévin et al., 2016) which is consistent with our data for some pathways.

We observed only small changes in the central carbon metabolism in both salts. Metabolites involved in the central carbon metabolism such as pyruvate and 2-oxoglutarate were not significantly increased. Under anoxic conditions *Y. intermedia* has the capability to perform fermentation, anaerobic respiration or mixed-acid fermentation (Babujee et al., 2013). This results in genes included in the TCA-cycle being down-regulated which might explain the lack of significantly identified metabolites involved in this specific energy pathway.

However, we detected significant changes in the various amino acid pathways which were partly salt-dependent. Since amino acids are required for growth, the uptake of certain amino acids is a conserved response in Enterobacteria under anoxic conditions.

While, the roles of the individual amino acids are not clear (Babujee et al., 2013), it is known that several amino acids such as asparagine, aspartate and glutamine which were all increased under NaCl and MgSO_4_ supplemented conditions, can be deaminated at fast rates with the products being fed into the central intermediary nitrogen metabolism processes while also producing large amounts of free ammonium as a byproduct (Mendz and Hazell, 1995). One of the main processes involved in the nitrogen assimilation is the urea cycle. *Y. intermedia* does not have arginase activity and therefore does not possess the full suite of enzymes involved in the urea cycle indicating an incomplete urea cycle function found in most microorganisms with a few exceptions (Mendz and Hazell, 1996). More specifically, arginine is not converted into ornithine and urea due to the lack of arginase. However, an increased ornithine level was found under the tested salt conditions which suggests that the glutamine and glutamate pathway lead to its accumulation. While the role of the urea cycle in prokaryotes is still unclear, one of its possible functions is to regulate the nitrogen amount within the cell. Another one can be the regulation of the internal pH. Enteric bacteria have developed various strategies for protection against acid stress. For *Y. pseudotuberculosis* an aspartate-dependent acid survival system is described (Hu et al., 2010) which might be also prevalent in our strain as it originates from an acidic lake. The observed increase in aspartate might be due to a potential decreased activity of the aspartate as part of the internal pH regulation to avoid additional increase of the pH as the above mentioned deamination reactions lead to the accumulation of excess ammonia. Another explanation could be the degradation of L-asparagine via asparaginase which is a deamidase producing ammonia and aspartate. To avoid excessive alkalination, ammonia is transformed into urea which is then removed from the cell by diffusion.

Furthermore, carbon starvation can lead to an increase of amino acids, presumably as the result of starvation-induced protein catabolism (Brauer et al., 2006). Similarly to halophilic bacterial isolates we were able to identify rare diaminoacids such N-acetyl-alpha-lysine and N-acetylornithine under salt stress (Joghee and Jayaraman, 2014). However, we found a salt-dependent response. For *E. coli*, the degradation of serine in order to maintain mobility has been described (Douarche et al., 2009). This might also be the case for *Y. intermedia* MASE-LG-1. Contradictory to our findings, it has been previously described that certain amino acids including leucine, tryptophan or methionine are depleted under salt stress (Sévin et al., 2016). In our study a heterogeneous response was detected for many amino acid pathway end products which also differed under NaCl stressed condition compared to MgSO_4_ stressed samples. The general alterations in the amino acid metabolism might be explained by the accumulation of the compatible solute L-asparagine in both salts.

## Conclusion

The investigation of the stress response using a global metabolome approach unraveled the metabolic response of *Yersinia intermedia* MASE-LG-1 under salt stress. We observed that some responses were the same in both NaCl and MgSO_4_, such as production of L-asparagine and sucrose, but that there were salt-specific changes in other metabolites, such as tyrosine, anthranilate, DAHP involved in the amino acid metabolisms. These differences suggest that water activity alone cannot predict the biological response. Other properties of the involved ions might influence growth. In these experiments, the ionic strength of NaCl was 0.4 mol/l and MgSO_4_ was 2.8 mol/l. Ionic strength has previously been proposed as a potential barrier to life in sulfate salts (Fox-Powell et al., 2016). Alternatively, differences might have been caused by different biological effects of specific ions. For example, while Mg^2+^ is toxic in high concentrations, Mg^2+^ is the most abundant divalent cation in microorganisms, involved in stabilizing macromolecular complexes and membranes, neutralizing nucleic acids and nucleotides in the cytoplasm and phospholipid head groups and surface molecules (Groisman et al., 2014). We conclude that the metabolic responses of organisms to non-NaCl salts may elicit the same pathways as in NaCl, but that unique properties of ions ultimately produce unique biochemical responses to particular ionic environments.

## Author contributions

PS planned the study, performed experiments, data analysis, and wrote the manuscript. MB assisted in performing the cultivation experiments. KB-V, CM-E, FG, and PE contributed by discussion of the result, proof reading the manuscript. CC conceptualized the MASE project as a whole, helped conceive the study and writing the manuscript. All authors approved the final manuscript.

### Conflict of interest statement

The authors declare that the research was conducted in the absence of any commercial or financial relationships that could be construed as a potential conflict of interest.

## Supplementary material

Supplementary Figure 1 Principal Component Analysis (PCA) plot showing the multivariate variation among the three sample sets (control, NaCl stressed and MgSO4 stressed; n = 3) in terms of the obtained peaks.

Supplementary Table 1 List of identified and putatively identified lipids. Metabolite levels for each experimental group are shown. Levels are expressed as mean peak intensity relative to the mean peak intensity of the control group. Numbers indicated in bold indicates statistically significance (*p* < 0.05).
