# Supplementary figures and images for: Beyond Chloride Brines: Variable Metabolomic Responses in the Anaerobic Organism Yersinia intermedia MASE-LG-1 to NaCl and MgSO4 at Identical Water Activity
Source: Front Microbiol. 2018 Feb 27;9:335. doi: 10.3389/fmicb.2018.00335 (PMC5835128; doi:10.3389/fmicb.2018.00335)

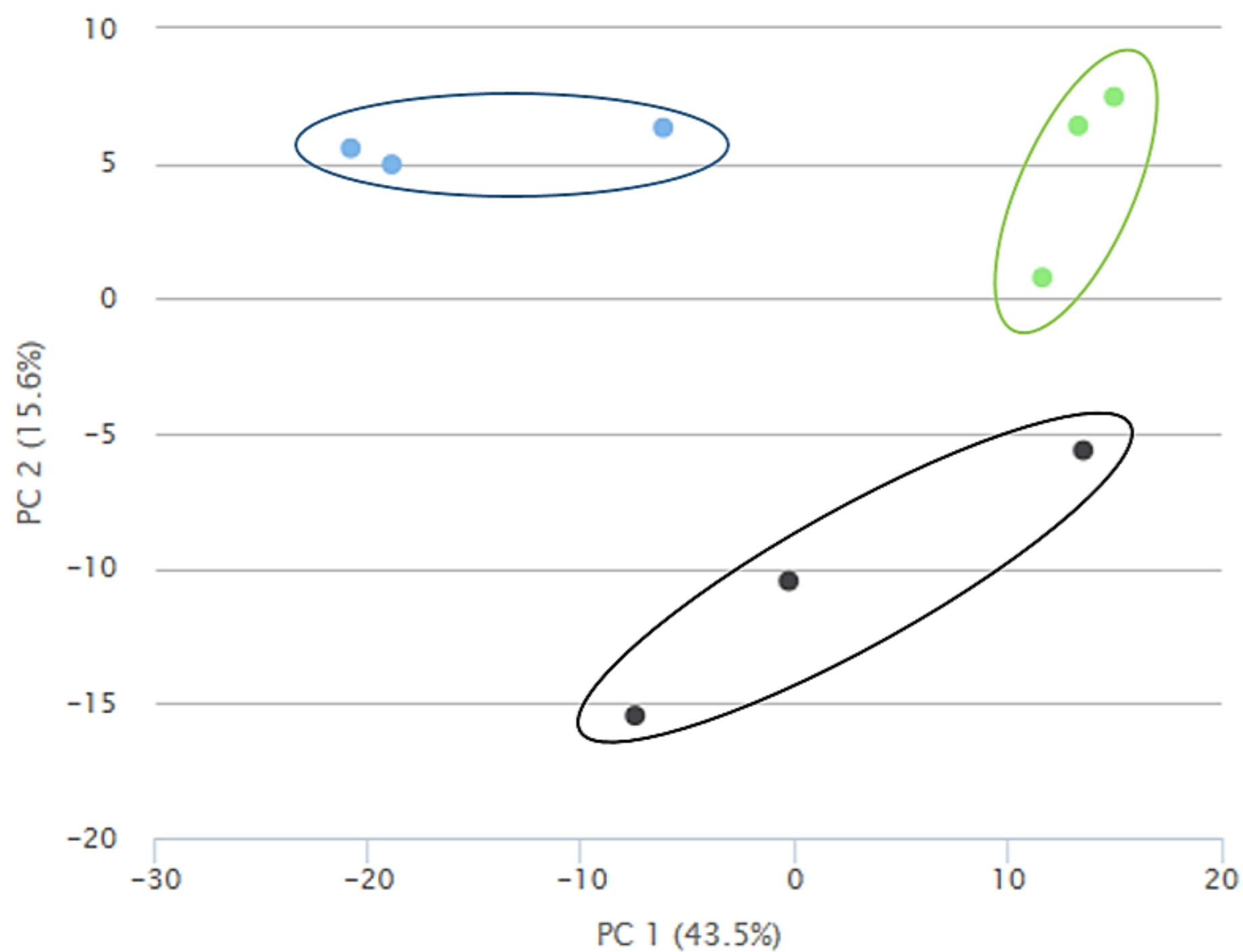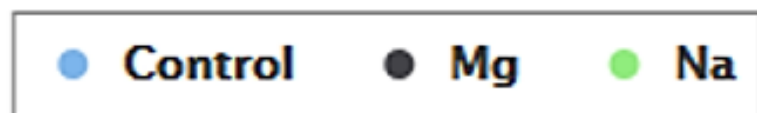

Supplement: Supplementary Figure 1 — Principal Component Analysis (PCA) plot showing the multivariate variation among the three sample sets (control, NaCl stressed and MgSO4 stressed; n = 3) in terms of the obtained peaks. [file Image1.PDF]
