# Supplementary material for: Beyond Chloride Brines: Variable Metabolomic Responses in the Anaerobic Organism Yersinia intermedia MASE-LG-1 to NaCl and MgSO4 at Identical Water Activity
Source: Front Microbiol. 2018 Feb 27;9:335. doi: 10.3389/fmicb.2018.00335 (PMC5835128; doi:10.3389/fmicb.2018.00335)
Supplement: Supplementary Table 1 — List of identified and putatively identified lipids. Metabolite levels for each experimental group are shown. Levels are expressed as mean peak intensity relative to the mean peak intensity of the control group. Numbers indicated in bold indicates statistically significance (p < 0.05). [file Table1.docx]

Supplementary Material

Beyond Chloride Brines: Variable Metabolomic Responses in the Anaerobic Organism, *Yersinia intermedia* MASE-LG-1, to NaCl and MgSO_4_ at Identical Water Activity

Petra Schwendner^1*^, Maria Bohmeier^2^, Petra Rettberg^2^, Kristina Beblo-Vranesevic^2^, Frédéric Gaboyer^3^, Christine Moissl-Eichinger^4,5^, Alexandra K. Perras^4,6^, Pauline Vannier^7^, Viggo T. Marteinsson^7^, Laura Garcia-Descalzo^8^, Felipe Gómez^8^, Moustafa Malki^9^, Ricardo Amils^9^, Frances Westall^3^, Andreas Riedo^10^, Euan P. Monaghan^10^, Pascale Ehrenfreund^10,11^, Patricia Cabezas^12^, Nicolas Walter^12^, Charles Cockell^1^

^1^ School of Physics and Astronomy, UK Center for Astrobiology, University of Edinburgh, Edinburgh, United Kingdom

^2^ Radiation Biology Department, Institute of Aerospace Medicine, German Aerospace Center (DLR), Cologne, Germany

^3^ Centre de Biophysique Moléculaire, Centre National de la Recherche Scientifique (CNRS), Orléans, France

^4^ Department of Internal Medicine, Medical University of Graz, Graz, Austria

^5^ BioTechMed-Graz, Graz, Austria

^6^ Department of Microbiology and Archaea, University of Regensburg, Regensburg, Germany

^7^ MATIS - Prokaria, Reykjavík, Iceland

^8^ Instituto Nacional de Técnica Aeroespacial - Centro de Astrobiología (INTA-CAB), Madrid, Spain

^9^ [Universidad Autónoma de Madrid](https://www.uam.es/ss/Satellite/en/home.htm) (UAM), Madrid, Spain

^10^ Leiden Observatory, Universiteit Leiden, Leiden, Netherland

^11^ Space Policy Institute, George Washington University, Washington DC, USA

^12^ [European Science Foundation (ESF), Strasbourg](http://www.esf.org/), France

*** Correspondence:**Petra Schwendner
petra.schwendner@ed.ac.uk

# Supplementary Data

Supplementary Table 1: List of identified and putatively identified lipids. Metabolite levels for each experimental group are shown. Levels are expressed as mean peak intensity relative to the mean peak intensity of the control group. Numbers indicated in bold indicates statistically significance (p < 0.05).

| **Map** | **Metabolite** | **Formula** | **Pathway** | **C** | **MgSO_4_** | **NaCl** |
| --- | --- | --- | --- | --- | --- | --- |
| Lipids:  Polyketides | [Fv] Spinochalcone C | C_25_H_26_O_3_ | Flavonoids | **0.00** | **2.52** | **9.65** |
| Lipid  Metabolism | **Taurine** | C_2_H_7_NO_3_S | Bile acid biosynthesis Taurine and hypotaurine metabolism | 1.00 | 1.37 | 0.60 |
|  | 2-C-Methyl-D-erythritol 4-phosphate | C_5_H_13_O_7_P | Biosynthesis of steroids | 1.00 | 2.00 | **5.74** |
|  | Tetradecanoic acid | C_14_H_28_O_2_ | Fatty acid biosynthesis | 1.00 | 0.66 | 0.54 |
|  | Dodecanoic acid | C_12_H_24_O_2_ | Fatty acid biosynthesis | 1.00 | 0.48 | 0.39 |
|  | Decanoic acid | C_10_H_20_O_2_ | Fatty acid biosynthesis | 1.00 | 0.45 | 0.39 |
|  | (9Z)-Hexadecenoic acid | C_16_H_30_O_2_ | Fatty acid biosynthesis | 1.00 | 0.04 | 0.07 |
|  | Octadecanoic acid | C_18_H_36_O_2_ | Fatty acid biosynthesis Biosynthesis of unsaturated fatty acids | 1.00 | 0.35 | 0.19 |
|  | Hexadecanoic acid | C_16_H_32_O_2_ | Fatty acid biosynthesis Fatty acid metabolism Biosynthesis of unsaturated fatty acids | 1.00 | 0.22 | 0.17 |
|  | Diethanolamine | C_4_H_11_NO_2_ | Glycerophospholipid metabolism | 1.00 | 2.06 | 1.55 |
|  | Triethanolamine | C_6_H_15_NO_3_ | Glycerophospholipid metabolism | 1.00 | **0.50** | 1.44 |
|  | **sn-glycero-3-Phosphocholine** | C_8_H_20_NO_6_P | Glycerophospholipid metabolism Ether lipid metabolism | 1.00 | 2.93 | 3.44 |
|  | sn-glycero-3-Phosphoethanolamine | C_5_H_14_NO_6_P | Glycerophospholipid metabolism Ether lipid metabolism | 1.00 | 0.74 | 0.23 |
|  | **Choline phosphate** | C_5_H_14_NO_4_P | Glycerophospholipid metabolism Glycine, serine and threonine metabolism | 1.00 | 0.41 | 1.67 |
|  | **(R)-3-Hydroxybutanoate** | C_4_H_8_O_3_ | Synthesis and degradation of ketone bodies Butanoate metabolism | 1.00 | 1.41 | 1.63 |
| Lipids:  Fatty Acyls | 9-Oxononanoic acid | C_9_H_16_O_3_ | alpha-Linolenic acid metabolism | 1.00 | 0.50 | 0.37 |
|  | [FA amino(12:0)] 12-amino-dodecanoic acid | C_12_H_25_NO_2_ | Amino Fatty Acids | 1.00 | 0.82 | 0.95 |
|  | [FA amino(13:0)] 13-amino-tridecanoic acid | C_13_H_27_NO_2_ | Amino Fatty Acids | 1.00 | 0.72 | 1.05 |
|  | [FA amino(11:0)] 11-amino-undecanoic acid | C_11_H_23_NO_2_ | Amino Fatty Acids | 1.00 | 0.79 | 1.30 |
|  | (S)-2-Aminobutanoate | C_4_H_9_NO_2_ | Amino fatty acids | 1.00 | 1.10 | 0.97 |
|  | [FA (7:0/2:0)] Heptanedioic acid | C_7_H_12_O_4_ | Biotin metabolism | 1.00 | 0.00 | 0.00 |
|  | [FA dimethyl,amino,tri-hydrox] 1-dimethylamino-9S,11R,15S-trihydroxy-5Z,13E-prostadiene | C_22_H_41_NO_3_ | Eicosanoids | 1.00 | 0.40 | 0.32 |
|  | [FA oxo,hydroxy(4:0)] 9-oxo-15S-hydroxy-5Z,10Z,13E,  17Z-prostatetraenoic acid | C_20_H_28_O_4_ | Eicosanoids | 1.00 | 0.63 | 0.61 |
|  | [FA hydroxy(20:2)] 11R-hydroxy-12E,14Z-eicosadienoic acid | C_20_H_36_O_3_ | Eicosanoids | 1.00 | 0.00 | 0.00 |
|  | [FA (8:0)] octanoic acid | C_8_H_16_O_2_ | Fatty acid biosynthesis | 1.00 | 0.32 | 0.26 |
|  | [FA (18:1)] 9Z-octadecenoic acid | C_18_H_34_O_2_ | Fatty acid biosynthesis__Biosynthesis of unsaturated fatty acids | 1.00 | 0.06 | 0.07 |
|  | [FA oxo(18:3)] 4-oxo-9Z,11E,  13E-octadecatrienoic acid | C_18_H_28_O_3_ | Fatty Acids and Conjugates | 1.00 | 0.69 | 0.41 |
|  | [FA oxo(8:0)] 3-oxo-octanoic acid | C_8_H_14_O_3_ | Fatty Acids and Conjugates | 1.00 | 0.82 | **0.62** |
|  | [FA (4:2/3:0)] 2-butyl-2-propenoic acid | C_7_H_12_O_2_ | Fatty Acids and Conjugates | 1.00 | 0.54 | 0.46 |
|  | [FA oxo(16:0)] 3-oxo-hexadecanoic acid | C_16_H_30_O_3_ | Fatty Acids and Conjugates | 1.00 | 0.22 | 0.22 |
|  | [FA (8:1)] 2Z-octenoic acid | C_8_H_14_O_2_ | Fatty Acids and Conjugates | 1.00 | **0.56** | **0.42** |
|  | [FA hydroxy(18:0)] 2S-hydroxy-octadecanoic acid | C_18_H_36_O_3_ | Fatty Acids and Conjugates | 1.00 | 0.24 | 0.24 |
|  | Nonanoic acid | C_9_H_18_O_2_ | Fatty Acids and Conjugates | 1.00 | 0.49 | 0.37 |
|  | [FA hydroxy(6:0)] 4-hydroxy-hexanoic acid | C_6_H_12_O_3_ | Fatty Acids and Conjugates | 1.00 | **1.48** | 1.27 |
|  | [FA (11:0)] undecanoic acid | C_11_H_22_O_2_ | Fatty Acids and Conjugates | 1.00 | 0.41 | 0.34 |
|  | [FA oxo(8:1)] 5-oxo-7-octenoic acid | C_8_H_12_O_3_ | Fatty Acids and Conjugates | 1.00 | 0.48 | 0.34 |
|  | [FA hydroxy(11:1)] 2-hydroxy-10-undecenoic acid | C_11_H_20_O_3_ | Fatty Acids and Conjugates | 1.00 | 0.34 | 0.26 |
|  | [FA (7:0)] heptanoic acid | C_7_H_14_O_2_ | Fatty Acids and Conjugates | 1.00 | **0.53** | **0.44** |
|  | Pentanoate | C_5_H_10_O_2_ | Fatty Acids and Conjugates | 1.00 | 0.63 | 0.97 |
|  | [FA trihydroxy(4:0)] 2,2,4-trihydroxy-butanoic acid | C_4_H_8_O_5_ | Fatty Acids and Conjugates | 1.00 | 1.63 | 1.49 |
|  | 2-Amino-9,10-epoxy-8-oxodecanoic acid | C_10_H_17_NO_4_ | Fatty Acids and Conjugates | 1.00 | 0.62 | 0.29 |
|  | [FA dioxo(8:0)] 4,7-dioxo-octanoic acid | C_8_H_12_O_4_ | Fatty Acids and Conjugates | 1.00 | 0.45 | 0.40 |
|  | [FA (17:0)] heptadecanoic acid | C_17_H_34_O_2_ | Fatty Acids and Conjugates | 1.00 | 0.24 | 0.18 |
|  | [FA methyl(18:0)] 11R,12S-methylene-octadecanoic acid | C_19_H_36_O_2_ | Fatty Acids and Conjugates | 1.00 | 0.01 | 0.02 |
|  | [FA methyl(14:0)] 12-methyl-tetradecanoic acid | C_15_H_30_O_2_ | Fatty Acids and Conjugates | 1.00 | 0.20 | 0.19 |
|  | [FA hydroxy(17:2)] 7-hydroxy-10E,16-heptadecadien-8-ynoic acid | C_17_H_26_O_3_ | Fatty Acids and Conjugates | 1.00 | 0.18 | 0.32 |
|  | (9Z)-Tetradecenoic acid | C_14_H_26_O_2_ | Fatty Acids and Conjugates | 1.00 | 0.17 | 0.19 |
|  | omega-Cyclohexylundecanoic acid | C_17_H_32_O_2_ | Fatty Acids and Conjugates | 1.00 | 0.03 | 0.04 |
|  | [FA (6:0)] O-hexanoyl-R-carnitine | C_13_H_25_NO_4_ | Fatty acyl carnitines | 1.00 | 0.89 | 0.79 |
|  | O-Butanoylcarnitine | C_11_H_21_NO_4_ | Fatty acyl carnitines | 1.00 | **1.37** | **1.85** |
|  | [FA hydroxy(9:1)] 4-hydroxy-2-nonenal | C_9_H_16_O_2_ | Fatty aldehydes | 1.00 | 0.53 | **0.35** |
|  | [FA oxo(6:0)] N-(3-oxo-hexanoyl)-homoserine lactone | C_10_H_15_NO_4_ | Fatty amides | 1.00 | 1.49 | 1.12 |
|  | Pentanamide | C_5_H_11_NO | Fatty amides | 1.00 | 0.80 | **0.56** |
|  | Dodecanamide | C_12_H_25_NO | Fatty amides | 1.00 | 1.08 | 0.61 |
|  | [FA hydroxy(5:2/20:4)] N-(5-hydroxy-pentyl)-5Z,8Z,11Z,14Z-eicosatetraenoyl amine | C_25_H_43_NO_2_ | Fatty amides | 1.00 | 0.49 | 0.34 |
|  | N-Butyryl-L-homoserine lactone | C_8_H_13_NO_3_ | Fatty amides | 1.00 | **0.78** | 0.75 |
|  | N-Heptanoylhomoserine lactone | C_11_H_19_NO_3_ | Fatty amides | 1.00 | 0.79 | **0.58** |
|  | [FA oxo(5:1/5:0/4:0)] 1R,2R)-3-oxo-2-(2'Z-pentenyl)-cyclopentanebutanoic acid | C_14_H_22_O_3_ | Octadecanoids | 1.00 | 0.23 | 0.11 |
|  | Hexanoic acid | C_6_H_12_O_2_ | Oxidation of Long Chain Fatty Acids | 1.00 | 0.69 | 0.60 |
| Lipids: Glycero-lipids | MG(0:0/14:0/0:0) | C_17_H_34_O_4_ | Monoradylglycerols | 1.00 | 0.64 | 0.40 |
|  | [GL (18:0)] 1-octadecanoyl-rac-glycerol | C_21_H_42_O_4_ | Monoradylglycerols | 1.00 | 0.67 | 0.27 |
| Lipids: Glycero-phospho-lipids | [GP (18:0/20:4)] 1-octa-decanoyl-2-(5Z,8Z,11Z,14Z-eicosatetraenoyl)-sn-glycero-3-phosphate (ammonium salt) | C_41_H_73_O_8_P | Glycerophosphates | 1.00 | 0.87 | 0.47 |
|  | [PC (14:0/18:2)] 1-tetradecanoyl-2-(9Z,12Z-octadecadienoyl)-sn-glycero-3-phosphocholine | C_40_H_76_NO_8_P | Glycerophosphocholines | 1.00 | 0.19 | 0.09 |
|  | [PC (14:0/16:1)] 1-tetradecanoyl-2-(9Z-hexadecenoyl)-sn-glycero-3-phosphocholine | C_38_H_74_NO_8_P | Glycerophosphocholines | 1.00 | 0.19 | 0.12 |
|  | [PC (14:1)] 1-(9Z-tetradecenoyl)-sn-glycero-3-phosphocholine | C_22_H_44_NO_7_P | Glycerophosphocholines | 1.00 | 0.02 | 0.06 |
|  | PC(14:1(9Z)/15:0) | C_37_H_72_NO_8_P | Glycerophosphocholines | 1.00 | 0.02 | 0.07 |
|  | [PE (16:0/18:2)] 1-hexadecanoyl-2-(9Z,12Z-octadecadienoyl)-sn-glycero-3-phosphoethanolamine | C_39_H_74_NO_8_P | Glycerophosphoethanolamines | 1.00 | 0.04 | 0.09 |
|  | [PE (18:1)] 1-(9Z-octadecenoyl)-sn-glycero-3-phosphoethanolamine | C_23_H_46_NO_7_P | Glycerophosphoethanolamines | 1.00 | 0.00 | 0.07 |
|  | [PE (16:0)] 1-hexadecanoyl-sn-glycero-3-phosphoethanolamine | C_21_H_44_NO_7_P | Glycerophosphoethanolamines | 1.00 | 0.00 | 0.04 |
| Lipids: Polyketides | [PK] Chrysophanol | C_15_H_10_O_4_ | chrysophanol biosynthesis | 1.00 | **0.23** | 0.54 |
| Lipids: Prenols | [PR] Iridotrial | C_10_H_14_O_3_ | Isoprenoids | 1.00 | 2.17 | 1.66 |
|  | [PR] Citronellyl acetate | C_12_H_22_O_2_ | Isoprenoids | 1.00 | 2.02 | 2.22 |
|  | [PR] (+)-15-nor-4-thujopsen-3-one | C_14_H_22_O | Isoprenoids | 1.00 | 0.36 | 0.24 |
|  | [PR] 1,13-Dihydroxy-herbertene | C_15_H_22_O_2_ | Isoprenoids | 1.00 | 0.27 | 0.20 |
|  | [PR] 1'-Hydroxy-4-keto-gamma-carotene glucoside/ 1'-OH-4-Keto-gamma-carotene glucoside/ (Carotenoid K-G) | C_47_H_70_O_7_ | Isoprenoids | 1.00 | 0.51 | 0.68 |
|  | [PR] Perillyl aldehyde | C_10_H_14_O | Limonene and pinene degradation | 1.00 | 0.32 | 0.19 |
| Lipids: Sphingo-lipids | [SP (14:0/2:0)] tetradecasphinga-4E,6E-dienine | C_14_H_27_NO_2_ | Sphingoid bases | 1.00 | 0.92 | **0.66** |
|  | [SP methyl(13:0/13:0/2:0)] methyl 3-(13,13-dibromotrideca-1E,12-dienyl)-2H-azirine-2S-carboxylate | C_17_H_25_NO_2_Br_2_ | Sphingoid bases | 1.00 | 0.80 | 0.37 |
| Lipids: Sterol lipids | [ST (3:2/4:0/3:0)] (5Z,7E)-(1S,3R)-11-(3-acetoxy-1-propynyl)-9,10-seco-5,7,9(11),10(19)-cholestatetraene-1,3,25-triol | C_32_H_46_O_5_ | Secosteroids | 1.00 | 0.73 | 0.38 |
|  | [ST hydroxy,methyl(4:0)] (22E)-(8S)-3-hydroxy-22-methyl-9,10-seco-1,3,5(10),22-cholestatetraen-9-one | C_28_H_42_O_2_ | Secosteroids | 1.00 | 0.13 | 0.10 |
|  | [ST (6:0/6:0/3:0)] (5Z,7E,22E,24E,26E)-(1S,3R)-26a,26b-dihomo-27-nor-9,10-seco-5,7,10(19),22,24,26(26a)-cholestahexaene-1,3,26b-triol | C_28_H_40_O_3_ | Secosteroids | 1.00 | 0.20 | 0.15 |
